# Peripheral blood, lung and brain gene signatures in recovered and deceased patients with COVID-19

**DOI:** 10.1007/s40203-025-00450-1

**Published:** 2025-10-22

**Authors:** Eric Twum, Ancha Baranova, Aman Ullah

**Affiliations:** 1 School of Systems Biology, George Mason University, Fairfax, VA 22030 USA; 2 Research Centre for Medical Genetics, Moscow, Russia 115478

**Keywords:** COVID-19, Recovery, Severity, Mitochondria, Oxidative phosphorylation, Telomere lengthening, Interleukin-6, Ubiquitin, Anosmia

## Abstract

COVID-19 is a multi-system disease that affects not only the respiratory system but also the neurological, hematological, and immune cells and tissues. The understanding of how the virus infiltrates and impacts a diverse set of human cells is still incomplete. This work examined bulk RNA-seq data from recovered COVID-19 patients (COVID-19 survivors with or without post-acute complications) to uncover the persisting perturbations in biological pathways. The investigation also analyzed the transcriptomic signatures in the lungs and brain tissues of deceased patients during the acute phase of infection to understand COVID-19 impact on these organs. In recovered COVID-19 patients, the severity of acute COVID-19 influenced the subsequent disturbance of gene expression patterns in the post-acute disease phase. Gene expression patterns in the lungs and brain tissues of deceased COVID-19 patients correlate with the diverse pulmonary and neurological symptoms observed in these patients. We have detected significant alterations in the expression of mitochondrial genes, such as COX7C, COX7A2, ATP5PO, ATP5F1C and NDUFB8, which reflect a compensatory upregulation in response to viral suppression of mitochondrial function. This COVID-19 induced response persists up to 24 weeks post-infection, suggesting a sustained effort of the host to mitigate mitochondrial dysfunction. Additionally, in critical and severe COVID-19 cases, gradual upregulation of UBE3A expression was detected, which previously has been linked to favorable COVID-19 outcomes. Long-term anosmia related SHH gene was upregulated in recovered critically-ill COVID-19 patients. IL6 emerged as a central hub gene consistently expressed in both lung and brain tissues of deceased COVID-19 patients, underscoring its role in driving the inflammatory pathophysiology of COVID-19 within these organs. Collectively, our study emphasizes IL6 as a central cytokine driving the inflammatory response in both lung and brain tissues, and demonstrates the virus’s capacity to induce long-lasting changes in nuclear-encoded mitochondrial gene expression in the blood of recovered patients. Our findings have significant implications for developing targeted interventions for post-COVID-sequelae.

**Supplementary Information:**

The online version contains supplementary material available at 10.1007/s40203-025-00450-1.

## Introduction

The coronavirus disease 2019 (COVID-19) is a novel infectious respiratory disease that has triggered a pandemic, emerging as the world’s foremost public health emergency. As of 7 January 2024, the global tally surpassed 774 million confirmed cases and over seven million deaths. Worldwide, there was a 4% rise in new COVID-19 cases in the 28-day span from 11 December 2023 to 7 January 2024, totaling over 1.1 million new infections, compared to the preceding 28-day timeframe. Between 11 December 2023 and 7 January 2024, there was a significant uptick in COVID-19 related hospitalizations and intensive care unit (ICU) admissions, witnessing increases of 40% and 13% respectively, with more than 173,000 new hospitalizations and 1900 ICU admissions (World 2024).

Acute COVID-19 disease may range from mild to moderate and severe presentations and is defined as the period within 0–28 days post symptom onset, during which patients exhibited active SARS-CoV-2 infection and clinical symptoms. Patients with ambulatory illness may exhibit a variety of signs and symptoms such as fever, cough, sore throat, malaise, headache, muscle pain, nausea, vomiting, diarrhea, loss of taste and smell. They, however, do not develop shortness of breath, dyspnea on exertion, or abnormal imaging. Moderate illness is defined as evidence of lower respiratory disease during clinical assessment or imaging, with an SpO2 ≥ 94% on room air at sea level whilst individuals suffering from severe COVID-19 exhibit oxygen saturation (SpO2) of < 94% on room air at sea level, with arterial oxygen partial pressure to fractional inspired oxygen ratio (PaO2/FiO2) less than 300 mm Hg. Patients suffering from critical COVID-19 can encounter severe health complications such as acute respiratory distress syndrome, virus-triggered distributive (septic) shock, cardiac shock, an intense inflammatory response, thrombotic disease, and a worsening of pre-existing comorbid conditions (National Institutes of Health COVID-19 Treatment Guidelines; Chan et al. 2020; Yang et al. 2020). Although there is emerging data suggesting a connection between the severity of acute COVID-19 and the likelihood of developing persistent symptoms, the current body of research does not offer sufficient depth and breadth to robustly support this viewpoint. Addressing this gap is pivotal for accurately identifying risk factors associated with long-term symptomatology, thereby facilitating improved patient risk assessment and management.

Undoubtedly, the respiratory system stands as the predominant target of SARS-CoV-2’s impact (Huang et al. 2020). Upon macroscopic examination, the deceased patient’s lungs exhibit notable characteristics, appearing dense, solid, and severely congested, with regions ranging from sporadic patches to widespread firm areas. In most cases, the predominant histological observation is the presence of diffuse alveolar damage and fibrosis (Ackermann et al. 2020). On the other hand, COVID-19 is associated with a spectrum of central nervous system symptoms, including loss of smell and taste, headaches, dizziness, cognitive impairment, seizures, strokes, and encephalopathy, with varying degrees of severity observed in affected individuals (Caronna 2020)–(Uginet et al. 2021). The research byXu et al. (2024) summarizing various neurological imaging findings in COVID-19 patients across various studies (Xu et al. 2024) showed that ischemic stroke, white matter irregularities, hemorrhagic lesions, deep vein thrombosis and perfusion irregularities emerged as the key pathological signs observed in patients with neurological symptoms. Currently, there is a dearth of comprehensive studies linking differential gene expression signatures in COVID-19 patients to the host of pathophysiological abnormalities found the lung and brain of acutely ill patients. Furthermore, there is very little research available on the comparative analysis of differential gene expression signatures across COVID-infected lung and brain tissue, representing a significant gap in our understanding of how the disease uniquely or otherwise impact these tissues.

Furthermore, current studies have indicated that SARS-CoV-2 has the capability to suppress the human body’s innate immune response, with mitochondria being among the initial defense mechanisms against SARS-CoV-2 infection (Burtscher et al. 2020). Ajaz and et al. (2021) conducted a study on the functional changes in mitochondria within live peripheral blood mononuclear cells (PBMCs) from COVID-19 patients, along with the resulting shifts in inflammatory pathways which revealed mitochondrial dysfunction and metabolic changes, including elevated glycolysis and increased mitokine levels in the PBMCs of these patients. Guarnieri et al. (Guarnieri et al. 2023), Medini et al. (Medini et al. 2021)have also suggested that after COVID-19 virus infection, there was a systemic host response followed by viral suppression of mitochondrial gene transcription and an ensuing elevated expression of DNA encoded oxidative phosphorylation (OXPHOS) genes resulting in a controlled rewiring of the OXPHOS machinery to glycolysis (Guarnieri et al. 2023; Medini et al. 2021). While these previous findings provide some understanding of OXHOS-related changes, there remains a critical void in our understanding of the temporal dynamics of these energy pathway alterations, particularly how long they persist beyond the acute phase of COVID-19. Hence, investigating how long these changes endure after the acute phase of the disease is essential for comprehending the long-term health consequences and potential interventions for affected individuals.

Hence, here we investigated: (1) gene pathways recovered COVID-19 patients to uncover the persisting perturbations in biological pathways (2) the impact of COVID-19 disease severity and how it affects gene expression signatures of the COVID-19 recovery trajectory and gene pathways and (3) characterized both common and unique gene expression patterns in the lungs and brain tissues of COVID-19 patients to elucidate their roles in the diverse pulmonary and neurological symptoms observed in these patients.

## Methods

### Datasets and data preprocessing

**Fig. 1:**
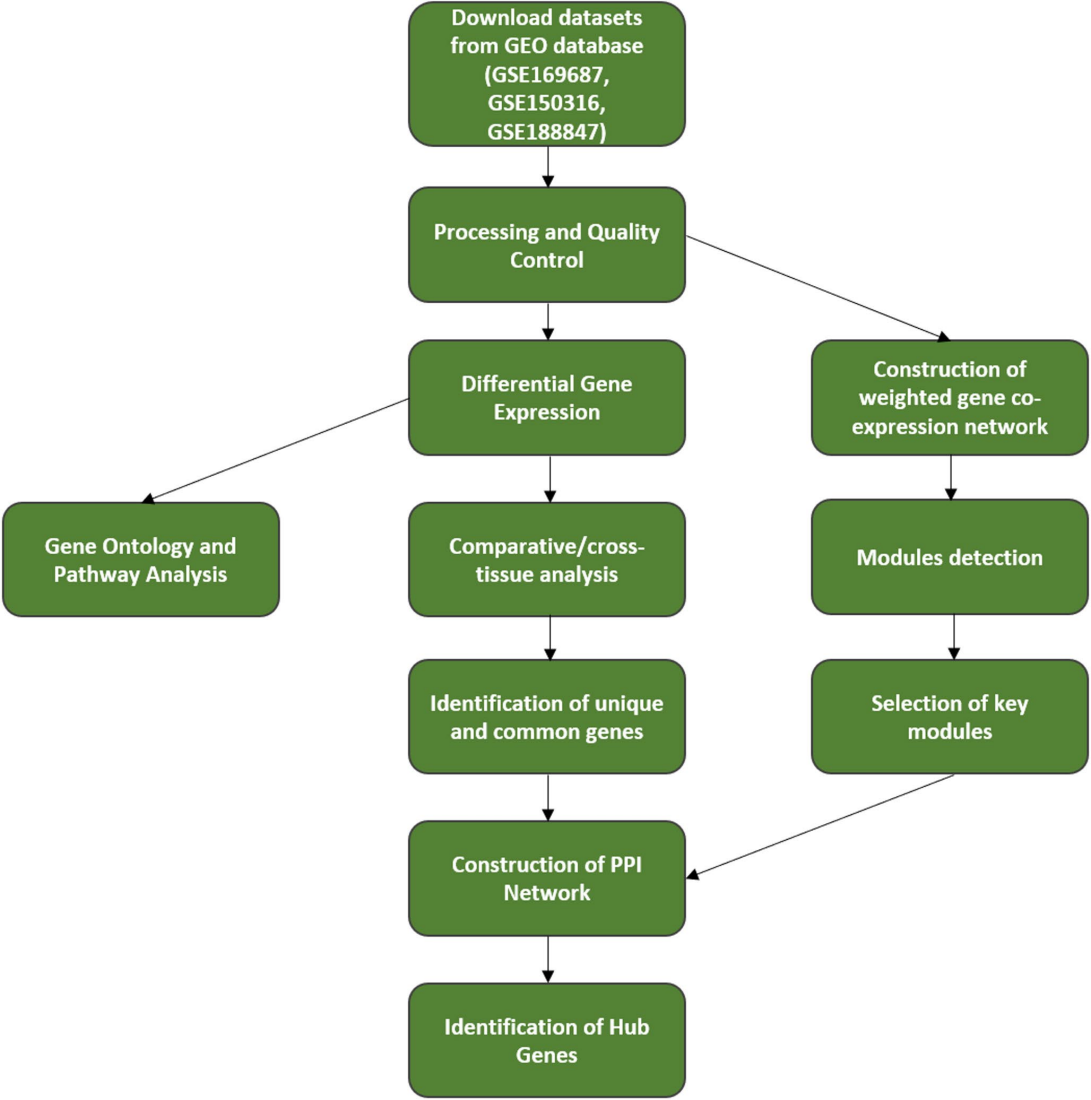
Flow chart of data sourcing, preparation, pre-processing, and analysis

First, high-throughput genomic sequences, clinical data, and annotated genetic information were retrieved from the GEO database, specifically datasets GSE169687, GSE150316, and GSE188847 (Ryan 2022; Desai 2020; https://www.ncbi.nlm.nih.gov/geo/query/acc.cgi?acc=GSE188847). These datasets encompassed bulk whole-genome sequencing information from various sources, including whole blood (GSE169687), lung epithelial tissue (GSE150316), and brain tissue (GSE188847) samples obtained from both normal subjects and individuals diagnosed with COVID-19. In this study, the term ‘recovered’ refers to patients who survived COVID-19 infection, regardless of whether they experienced persistent symptoms or post-acute complications. These individuals are thus considered ‘non-deceased COVID-19 patients. In all three datasets, the term ‘control’ refers to healthy or normal individuals with no known history of SARS-CoV-2 infection. A summary of the study datasets in the original data is summarized in Table1. In the initial data preprocessing steps, we excluded genes with zero counts across all samples to ensure robustness in subsequent analyses. The workflow for this study is presented in Fig. 1.

**Table 1:** Gene expression datasets used in the transcriptomic profiling of human blood, lung, and brain samples

GEO GSE_ID	Authors/Reference	Tissue Type	Number of Samples	Study Groups
GSE169687	Ryan et al. BMC Med (Ryan 2022)	Blood	**152**	Healthy controls, recovered patients (mild, moderate, severe, critical)
GSE150316	Desai et al. Nat Commun (Desai 2020)	Lung	**10**	Deceased COVID-19 patients, healthy controls
GSE188847	Lee et al. (Unpublished) 2021	Brain	**24**	Deceased COVID-19 patients, healthy controls

The original Raw data series was downloaded from GEO (corresponding to GSE ids: GSE169687; GSE150316; GSE188847)

### Differential gene expression

We used the “DSeq2” package (Love et al. 2014) within the R software environment identify genes displaying differential expression between normal subjects and COVID-19 patients across three post-infection timepoints – 12 weeks post-infection, 16 weeks-post infection and 24-weeks post infection. We also tested the associations between gene expression and patients with critical, severe, moderate and mild disease at the 12 weeks post-infection. All gene expression values were subjected to log2 transformation for normalization. Genes exhibiting |log2 fold change (FC)| >1 and a p-adjusted value of*p* < 0.05 were considered statistically significant. Notably, genes with smaller p-adjusted values were accorded higher rankings.

### Gene ontology (GO) and pathway analysis

To gain deeper insights into the biological significance of the identified key genes, we conducted Gene Ontology (GO) and Gene Set Enrichment Analyses (GSEA) using the R software packages “DOSE,” “org.Hs.eg.db,” and “enrichplot“(Carlson 2019; Yu 2023; Yu et al. 2015). For our study, we selected sets of differentially expressed genes as our test data, applying a significance threshold of*P* < 0.05. We identified statistically significant gene enrichment pathways, with a particular emphasis on “Biological Process” (BP) in the GO enrichment analysis.

### Identification of intersecting and unique DEGs

The R package “VennDiagram” serves as a powerful tool for visualizing and interpreting these intersections, providing insights into common or unique genes across multiple datasets (Chen and Boutros 2011). We used the “VennDiagram” package to identify shared genes expressed across different patient groups tissue tissues and patient groups, as well as genes uniquely expressed by each, to highlight their role as potential pathophysiological contributors in the context of the disease.

### Generation of protein–protein interaction (PPI) network for predicted genes

In this study, we established a Protein-Protein Interaction (PPI) network involving significant DEGs using the Search Tool for the Retrieval of Interacting Genes (STRING) database (https://string-db.org/cgi/input.pl; STRING-DB v12.0) (Szklarczyk 2019). To assess the prominence of interactions within this network, we ranked the target proteins based on their degree of connectivity using the Cytoscape plugin cytoHubba (Chin 2014). Subsequently, we imported the resultant protein interaction data for the target proteins into Cytoscape 3.9.1 software to construct a comprehensive PPI network (Shannon 2003).

### Construction of weighted gene expression Co-network

Weighted Gene Co-expression Network Analysis (WGCNA) has emerged as a pivotal tool for deciphering the complexities of gene expression data, particularly in the context of bulk RNA-sequencing. WGCNA constructs networks based on the pairwise correlation of gene expression profiles, assigning higher weights to gene pairs with stronger correlations. This approach not only accentuates significant relationships but also minimizes the impact of noise and spurious associations (Langfelder and Horvath 2008). The method clusters genes into modules, where genes within a module are more strongly correlated with each other than with genes outside the module. Each module is characterized by an eigengene that represents the module’s overall expression profile, facilitating the association between modules and external traits (Zhang and Horvath 2005). In this dissertation, WGCNA was utilized to identify functional modules enriched for genes that persist post-COVID across 12-, 16- and 24-week timepoints.

## Results

To comprehend the enduring effects of COVID-19, we aimed to surface persistent perturbations in gene pathways during post-infection recovery. Utilizing GEO dataset GSE169687 from the NCBI GEO database, we performed differential gene expression analysis to identify genes and biological pathways that remain impacted in recovered patients at 12-, 16-, and 24-weeks post-infection, shedding light on the lasting consequences of the disease. Specifically, we compared recovered COVID-19 patients at 12-, 16-, and 24-weeks post-infection to healthy controls.

### Differential gene expression analysis highlights distinct Temporal signatures in COVID-19 recovery

**Fig. 2:**
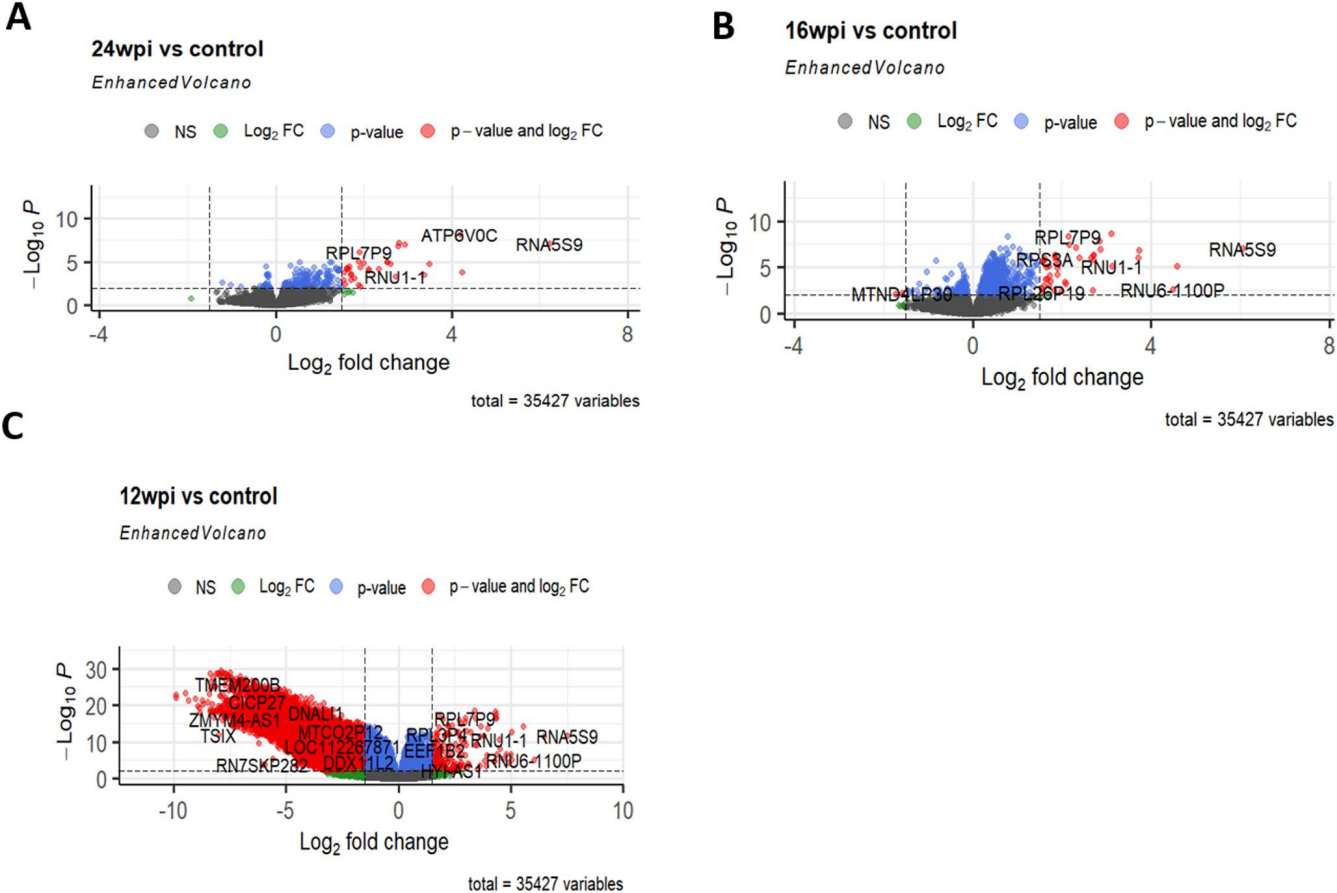
The distribution of differentially expressed genes (DEGs) at 12 weeks, 16 weeks and 24 weeks post COVID-19 infection between patients and healthy controls. **A**, **B**, **C**: Enhanced volcano plots of DEGs at **A** 12 weeks, **B** 16 weeks and **C** 24 weeks after COVID-19 recovery. The blue, green and gray dots indicate non-significant (NS) genes, whilst the red ones depict significant genes with both *p*-value and log2 fold change. There is a consistent pattern of *RNA59* being differentially expressed across different time points post-infection, suggesting it may play key role in the host response to COVID-19

When we analyzed the gene expression profiles of individuals at 12 weeks post-infection (12wpi) in comparison to the control group, we found that a higher proportion of genes (21%) were downregulated in the 12wpi group, in contrast to the upregulated genes (12%). This indicated a prevailing downregulation of gene expression in this particular group (12wpi). Conversely, when we examined the gene expression patterns of recovered patients at 16 weeks post-infection (16wpi) in comparison to the control group, there was a greater prevalence of upregulated genes (4.5%) compared to downregulated genes. This shift toward upregulation suggested a dynamic change in gene expression at this stage (16wpi). Similar to the 16wpi group, the gene expression profile of individuals at 24 weeks post-infection (24wpi) exhibited a higher percentage of upregulated genes (1.5%) than downregulated genes (0.3%) when compared to the control group. This consistent pattern of increased gene expression indicated a continued trend toward upregulation at the 24-week mark. The distribution of differentially expressed genes among 12wpi, 16wpi and 24wpi groups compared with control are shown in Fig. 2 and supplementary Figure S2, A-C.

To gain insights into the key significant differentially expressed genes across all patient groups in comparison to the control, we conducted an analysis focusing on the top 10 genes and visualized the results heatmaps (see supplementary data, Figure S1). The heatmaps revealed noteworthy enrichment of genes associated with various biological processes in COVID patients compared to controls across all time-points. Among these genes were *INSYN2B*,* PZP*,* CFLAR-AS1*,* RNA5S9*,* B2M*,* NEIL3*, along with several pseudogenes and non-coding RNA genes. Furthermore, distinct gene expression signatures were observed within the patient groups at different recovery time points. Specifically, the 12-week post-infection (12wpi) patient group exhibited a unique top ten differential gene expression profile that included the following genes: *INSYNB*,* IMPDH1P10*,* SEMA3F-AS1*,* PZP*,* CFLAR-AS1*,* C5orf58*,* LOC124903574*,* TMEM14EP*,* LINC01890*,* and RBM5-AS1* (see supplementary data, Figure. S1, A).

In contrast, genes such as *RNA5S9*,* RPL7B9*,* MIR3609*,* RPS3AP26*, and *RPL21P16* were commonly found among the top ten differentially expressed genes in both the 16-week post-infection (16wpi) and 24-week post-infection (24wpi) patient groups (see supplementary data, Figure S1, B-C).

### Alterations in nuclear-encoded OXPHOS genes persist beyond COVID-19 recovery

**Fig. 3:**
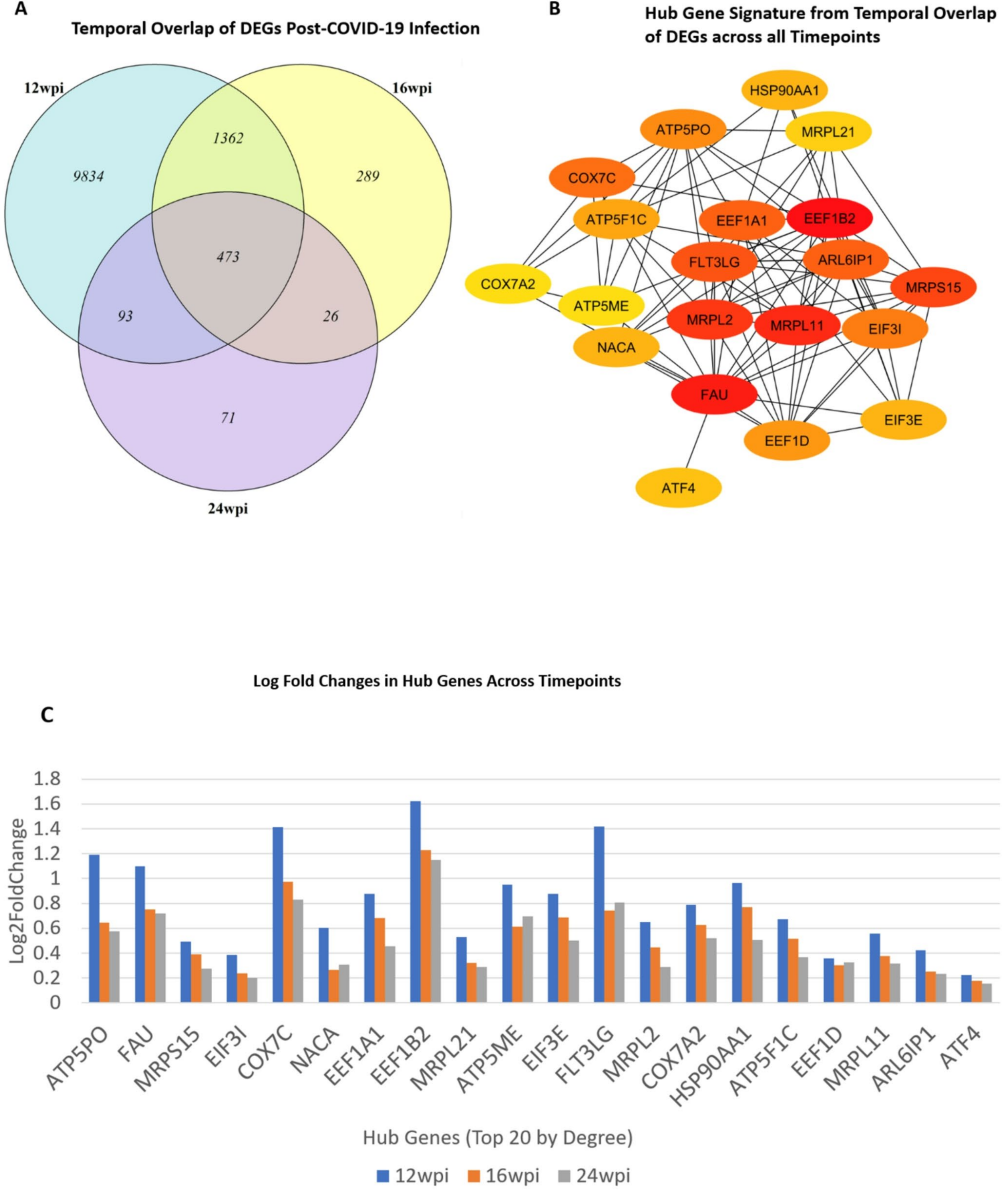
**A** Persisting gene expression post-COVID-19 infection. There are 9834 unique DEGs at 12 weeks, 289 unique DEGs at 16 weeks, 71 DEGs at 24 weeks and 473 DEGs common to all timepoints. **B** Protein-Protein Interaction (PPI) map of the hub genes (top 20 ranked by degree) from Intersecting DEGs across 12, 16 and 24 weeks from deceased COVID-19 patients. The hub gene signature revealed a distinctive gene signature associated with the mitochondrial electron transport chain. **C** Log fold changes of hub genes at 12-, 16 and 24-weeks post infection. With the exception of *NACA*,* ATP5ME*,* FLT3LG* and *EEF1D*, all hub genes showed a prevailing pattern of downregulation from 12 weeks post infection to 24 weeks post infection. The genes *NACA*, *ATP5ME*,* FLT3LG* and *EEF1D* were downregulated at 12 weeks and 16 weeks, but showed an increase in log fold changes at 24 weeks

To explore persisting genetic disruptions over 12-, 16-, and 24-weeks post-infection, we performed a thorough comparative study of DEGs at these specific intervals in two steps—identify common gene lists across all post-infection timepoints and detect hub gene signature that reveals gene disruption across these timepoints. This analysis revealed a distinct pattern of gene expression: 9,834 DEGs were unique to the 12-week mark, 289 to the 16-week mark, and 71 to the 24-week mark at the initial step. Additionally, we discovered that 473 genes were consistently differentially expressed across all these post-infection timepoints, highlighting a core set of genes impacted by the infection over time. (Fig. 3A). Subsequent to our initial analysis, we generated a PPI map focusing on the top 20 hub genes, ranked by their interaction degree, utilizing STRING. This approach was instrumental in identifying and characterizing the hub genes within this gene set, uncovering a distinctive gene signature linked to the mitochondrial electron transport chain (Fig. 3B). This finding is consistent with previous research highlighting the negative impact of SARS-Cov-19 virus on mitochondrial bioenergetics, thereby aiding in its persistence and propagation, leading to increased mitochondrial biogenesis, oxidative phosphorylation, and ATP production (Shin 2021). The variations in the expression patterns of the top 20 genes (by degree) across the post-recovery timepoints are depicted in Fig.3C. While most hub genes exhibited a consistent trend of downregulation from 12 to 24 weeks post-infection, there were notable exceptions. Specifically, the genes *NACA*, *ATP5ME*, *FLT3LG*, and *EEF1D* demonstrated increasing downregulation at 12 and 16 weeks, but interestingly, they exhibited an increase in log fold changes at the 24-week mark. For a complete set of all 473 genes expressed across all timepoints, see supplementary data Excel file Sheet S5.

Across the three post-infection timepoints overall, our analysis identified a total of 11,503 significantly upregulated genes and 2168 significantly downregulated genes for the 12wpi-control comparison, as well as 647 significantly downregulated genes for the 24wpi-control comparison (see supplementary data Excel file sheets S1, S2 and S3).

To explore temporal trends in biological activity during recovery, we performed enrichment map analysis comparing each patient group to healthy controls. At 12- and 16-weeks post-infection, patients exhibited upregulation of pathways related to telomere regulation, ribosomal RNA maturation, and antimicrobial humoral responses, while processes involving cyclin-dependent kinases, histone methylation, and synapse organization were downregulated. No significant changes were observed at 24 weeks. A detailed visualization of enrichment map clusters for the 12- and 16-week timepoints is provided in Supplementary Figure S5.

### Gene expression modules highlight key regulatory genes associated with 12 weeks post infection

**Fig. 4:**
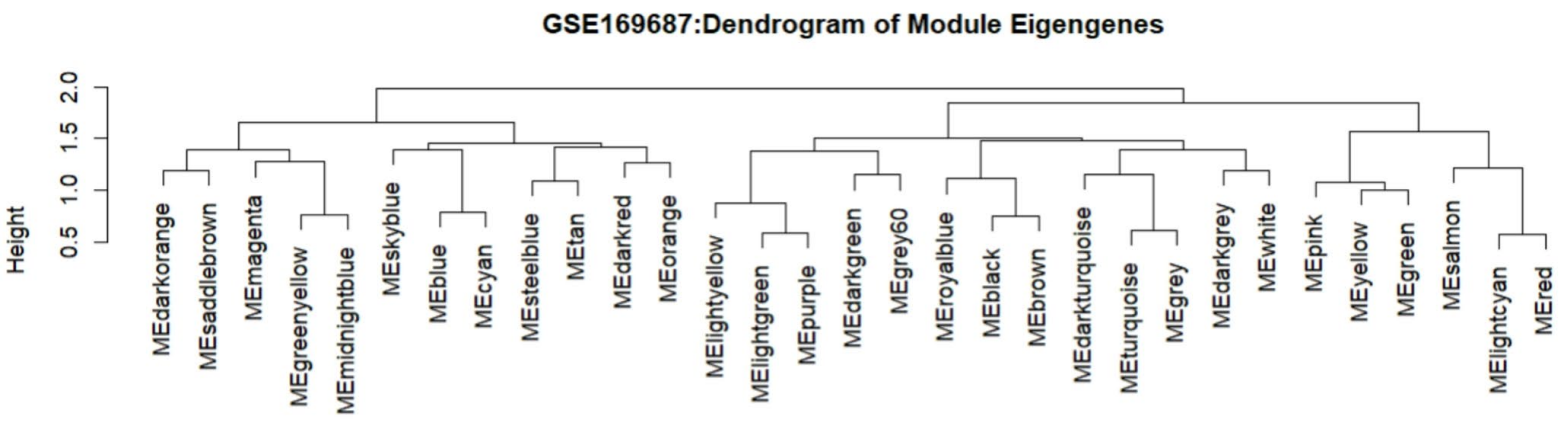
The cluster of modules eigengenes. The dendrogram illustrates the hierarchical clustering of module eigengenes, which represent the overall expression profiles of different modules. The y-axis indicates the dissimilarity (height) between clusters, with shorter heights denoting more similar modules. Each branch is labeled with a unique color representing a specific module

Modules or clusters, formed with a set of genes with correlated expression patterns, play a critical role in regulating tissues or organs. To identify potential gene clusters in blood tissue in response to SARS-CoV-2 recovery, we conducted WGCNA with suitable soft-thresholding power using normalized expression matrix obtained from GSE169687. Hierarchical cluster analysis with the average method indicated samples were well clustered and with only one outlier. Next, we analyzed network topology to choose the soft-thresholding power. The result showed that the softer threshold 9 corresponds to the lowest power, with a scale-free topology fit index of 0.80 and a relatively high average connectivity (see supplementary data, Figure S3 A-B). A total of 31 different color-coded co-expression modules were identified (Fig. 4).

**Fig. 5:**
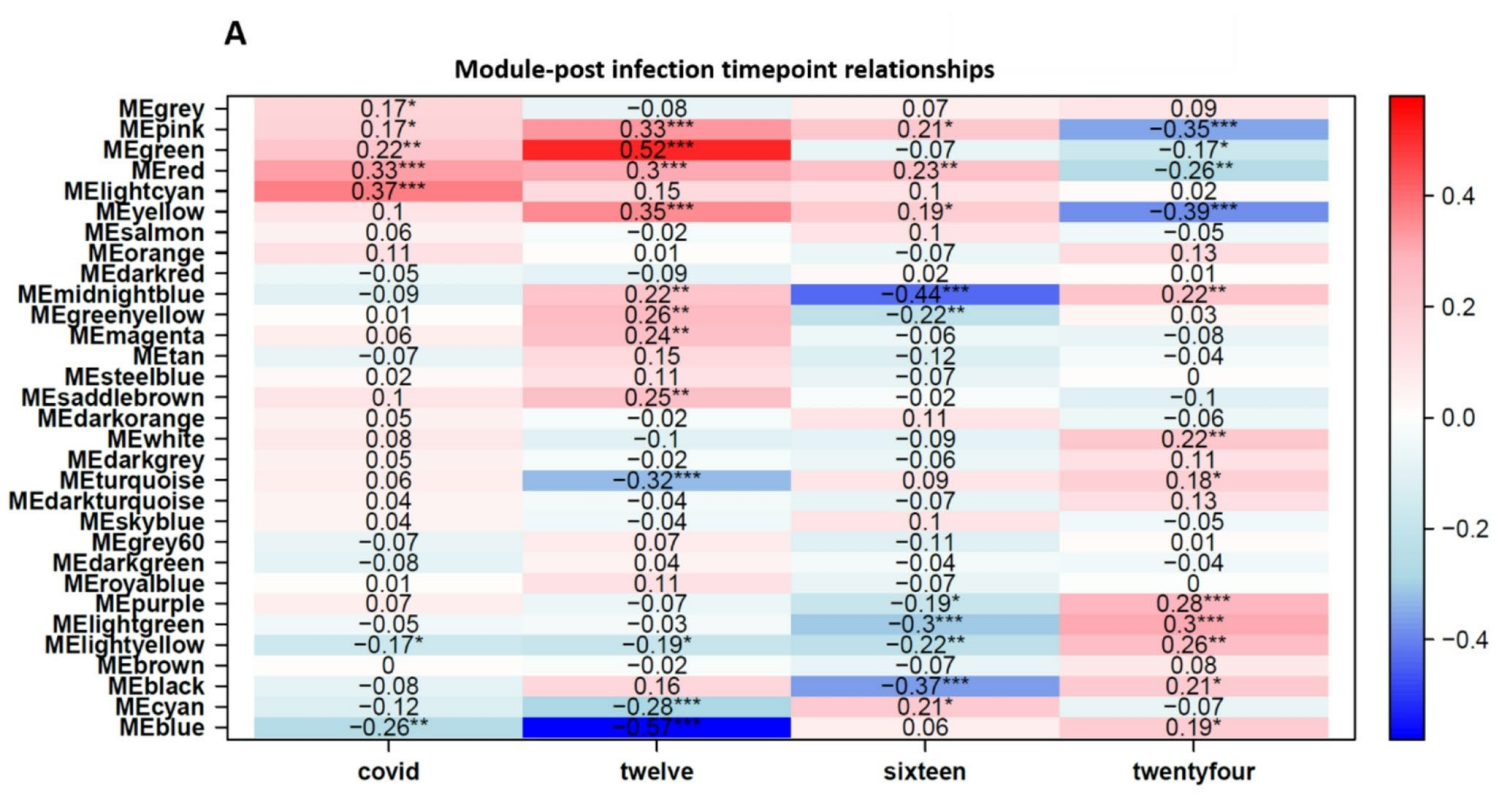
Gene significance and module membership of GSE169687. The association of modules and COVID-19 post infection timepoints were constructed. Each row refers to a module eigengene, whilst the second, third and fourth columns refer the post-infection timepoints. The first column represents the combined data for all post-infection timepoints as a single group

To determine the highest level of association significance, the correlation between the modules and the post-infection timepoints was analyzed. The cluster and correlation of all genes were depicted with heatmap (Fig. 5A). We observed that the ‘blue’ module, containing 5315 genes, showed the most significant correlation with 12-week post infection timepoint (Fig. 5A). To determine the highest level of association significance, the correlation between the modules and the post-infection timepoints was analyzed. We observed that the ‘pink’, ‘green’, ‘yellow’, ‘turquoise’ and ‘blue’ modules, containing 910, 1495, 2151, 9438 and 5315 genes respectively, indicated the most significant correlation across the post-infection timepoints. Further filtering of these modules showed *ARID4A and NDUFB8. UBE3A*,* KIF1A* and *FARP1* as the most significant genes for the ‘pink’, ‘green’, ‘yellow’, ‘turquoise’ and ‘blue’ modules respectively.

*ARID4A* is also upregulated post-infection at 12 weeks, but its expression is reduced at the 16-week mark. The *UBE3A*gene, which encodes an E3 ubiquitin-protein ligase, was found to be upregulated at 12 weeks post infection. This gene is part of the ubiquitin protein degradation system and previous research shows elevated ubiquitination contributes to protective immunity against severe SARS-CoV‐2 infection (Che et al. 2022). A mutation in the*UBE3A*has been identified as the cause of the Angelman Syndrome (AS), debilitating neurodevelopmental disorder that is characterized by motor dysfunction, intellectual disability, speech impairment, seizures and common features of autism spectrum disorders (ASDs) (Jiang et al. 1998). Remarkably, the case report of 14-year-old boy with AS who presented with severe COVID-19 symptoms in 2021 provides clinical evidence that patients with condition appear to be susceptible to a more severe form of COVID-19 (Lopes et al. 2021).

Remarkably, *NDUFB8* gene is also one of the persistently perturbed genes across all the post-infection timepoints and one that is overexpressed in recovered COVID-19 patients at 12 weeks post-infection but progressively and consistently exhibits a reduced positive fold change through week 16 up to week 24 (see supplementary data Excel file sheets S1, S2, S3 and S4). Interestingly, *NDUFB8*has been previously shown to be one of the host proteins that is targeted by the SARS-CoV-2 as part of host mitochondrial function manipulation (Guarnieri et al. 2022).

### COVID-19 acute disease severity impacts gene expression post-COVID recovery

Understanding how the severity of acute COVID-19 influences gene expression after recovery is crucial for patient risk assessment and management. To this end, we analyzed the GEO dataset GSE169687 to explore how disease severity correlates with gene expression patterns during the early recovery phase, uncovering valuable insights into the immediate consequences of COVID-19. Given that the 12wpi timepoint represents the closest recovery phase to the acute infection stage, we compared patients in this group who had experienced critical, severe, moderate, and mild COVID-19 with healthy individuals in terms of differential gene expression. We hypothesized that the earliest post-COVID recovery phase, 12 weeks post infection timepoint could still reveal the severity of COVID-19 experienced during the acute infection stage.

**Fig. 6:**
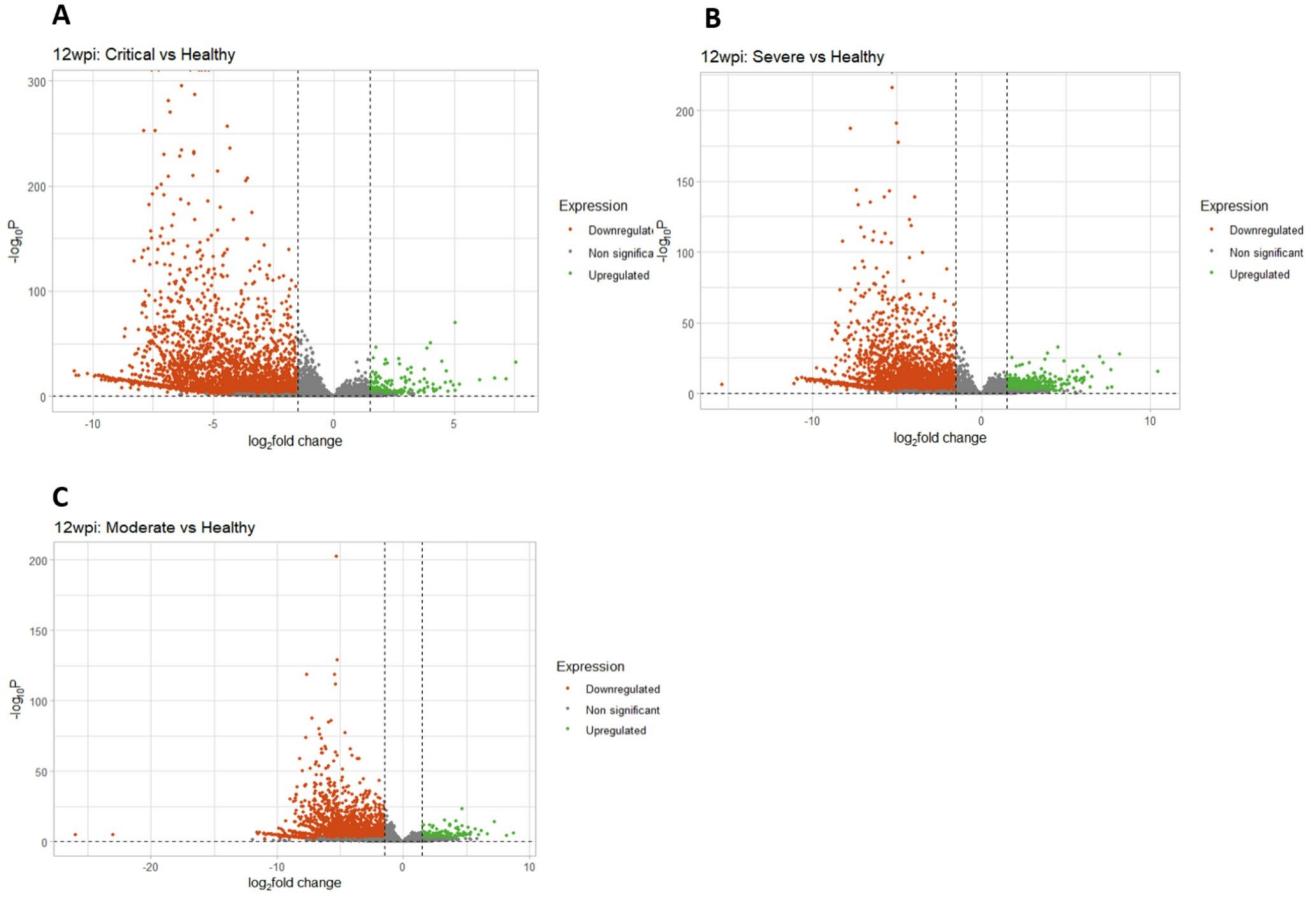
Volcano plots showing differential gene expression in recovered COVID-19 patients compared to healthy controls at 12wpi. Points in red represent downregulated genes, points in green represent upregulated genes, and points in gray represent genes that are not significantly differentially expressed. **A–C** Across all COVID severities, downregulation of genes is more common than upregulation when compared to healthy controls. The severity of the disease correlates with the number of significantly downregulated genes

Notably, among patients with critical, severe, and moderate COVID-19, We observed a decreasing pattern in the differential expression of downregulated genes, which corresponded to decreasing disease severity, as visually depicted in Fig. 6. Particularly, both critical and severe COVID-19 cases at 12wpi exhibited a shared suppression of genes primarily responsible for the detection of chemical stimuli involved in sensory perception of smell. Expectedly, we did not observe noticeable differences between the control group and patients who had experienced mild, moderate, severe, or critical COVID-19 at 16wpi or 24wpi.

**Fig. 7:**
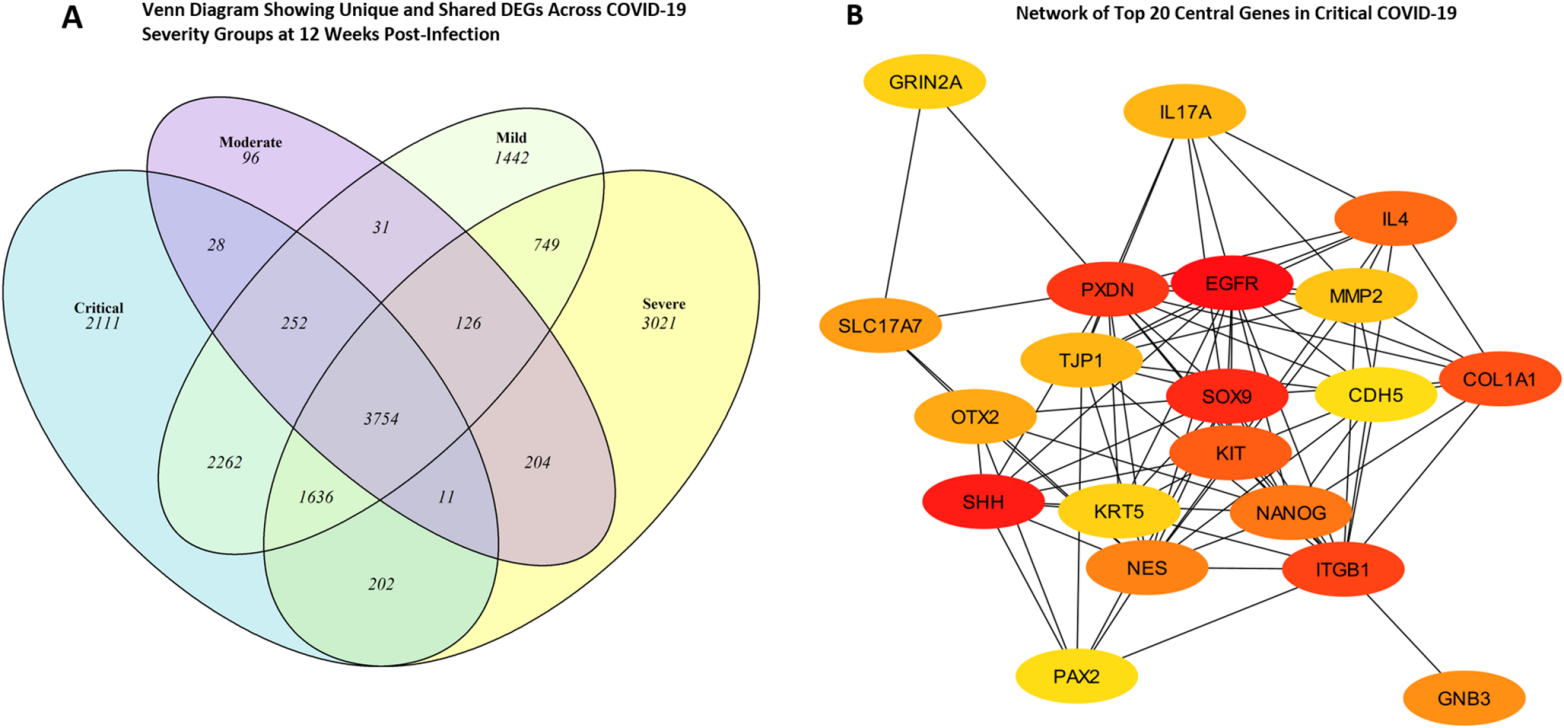
Unique Gene signatures in critical COVID-19 **A** 2111 DEGs were identified as uniquely persistently perturbed at 12 weeks post-infection in recovered patients that experienced COVID-19. **B** Further refinement (top 20 by degree) of the perturbed genes in recovered patients that had critical COVID revealed a unique gene signature with *EGFR* as a central node

**Fig. 8:**
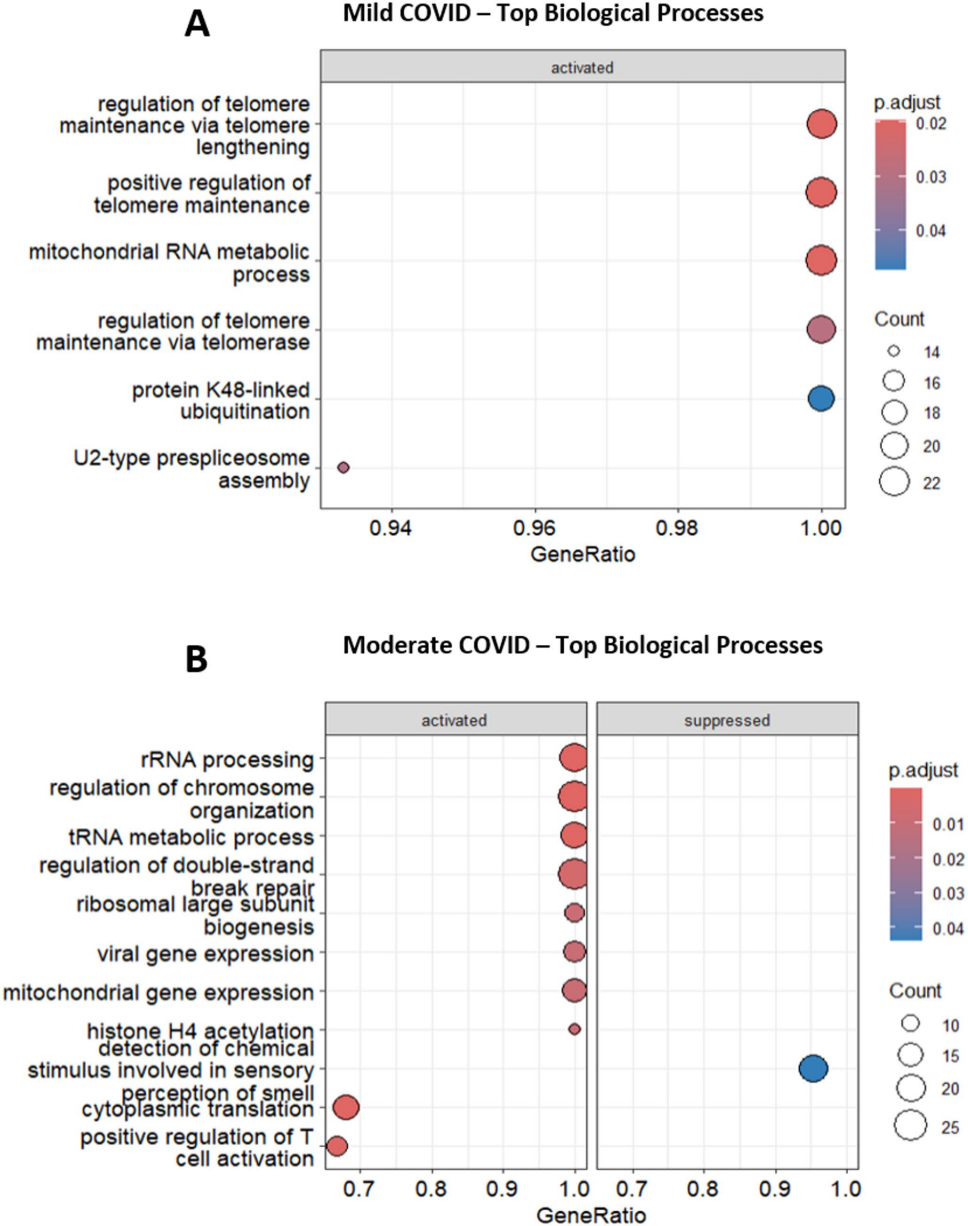
Dot plots showing GO enrichment analysis for biological processes in recovered COVD-19 patients at 12 weeks post infection compared to controls. The x-axis represents the GeneRatio, which is the ratio of genes in a particular GO term to the total number of genes involved in that process. The y-axis lists specific biological processes. The size of each dot indicates the count of genes involved (larger dots represent a higher count), and the color indicates the p-adjusted value (with darker colors representing higher significance) **A** Mild COVID: Biological processes focus on cellular maintenance and repair mechanisms, as indicated by processes related to telomere maintenance and mitochondrial function. The sensory perception of smell is notably affected in **B** moderate COVID-19 patients, aligning with clinical reports of anosmia in COVID-19 patients

To understand the genes central to all disease severities, as well as the genes unique to each disease severity, we again analyzed the DEGs for each disease criticality and teased out the distinct genes for each category (Fig. 7A). Because critical COVID-19 is more likely to result in dire medical complications and death (Chen and Boutros 2011), we focused on surfacing the hub genes associated with critical COVID-19, hoping to pinpoint crucial gene nodes that could be potential targets for therapeutic interventions, thereby reducing the likelihood of progression to critical COVID-19 and improving patient outcomes. Our analysis revealed the following genes (top 20 by degree) distinct to recovered patients at 12wpi timepoint who experienced critical COVID-19:*EGFR*,* SHH*,* SOX9*,* PXDN*,* ITGB1*,* COL1A1*,* KIT*,* IL4*,* NANOG*,* NES*,* GNB3*,* SLC17A7*,* OTX2*,* IL17A*,* TJP1*,* MMP2*,* GRIN2A*,* KRT5*,* PAX2* and *CDH5* (Fig. 8B) with *EGFR* as a central hub gene. All the hub genes with the exception of *ITGB1* were downregulated at 12 weeks in patients that experienced critical COVID-19 in the acute phase of the disease. Interestingly, *UBE3A* which emerged as the most significant gene from the ‘yellow’ module in the WGCNA analysis step was found to be upregulated at 12 weeks in critically and severely-ill COVID-19 patients who had recovered, but it was less expressed in the former group than the latter. This finding is consistent with the protective role of this gene in COVID-19 infection.

**Fig. 9:**
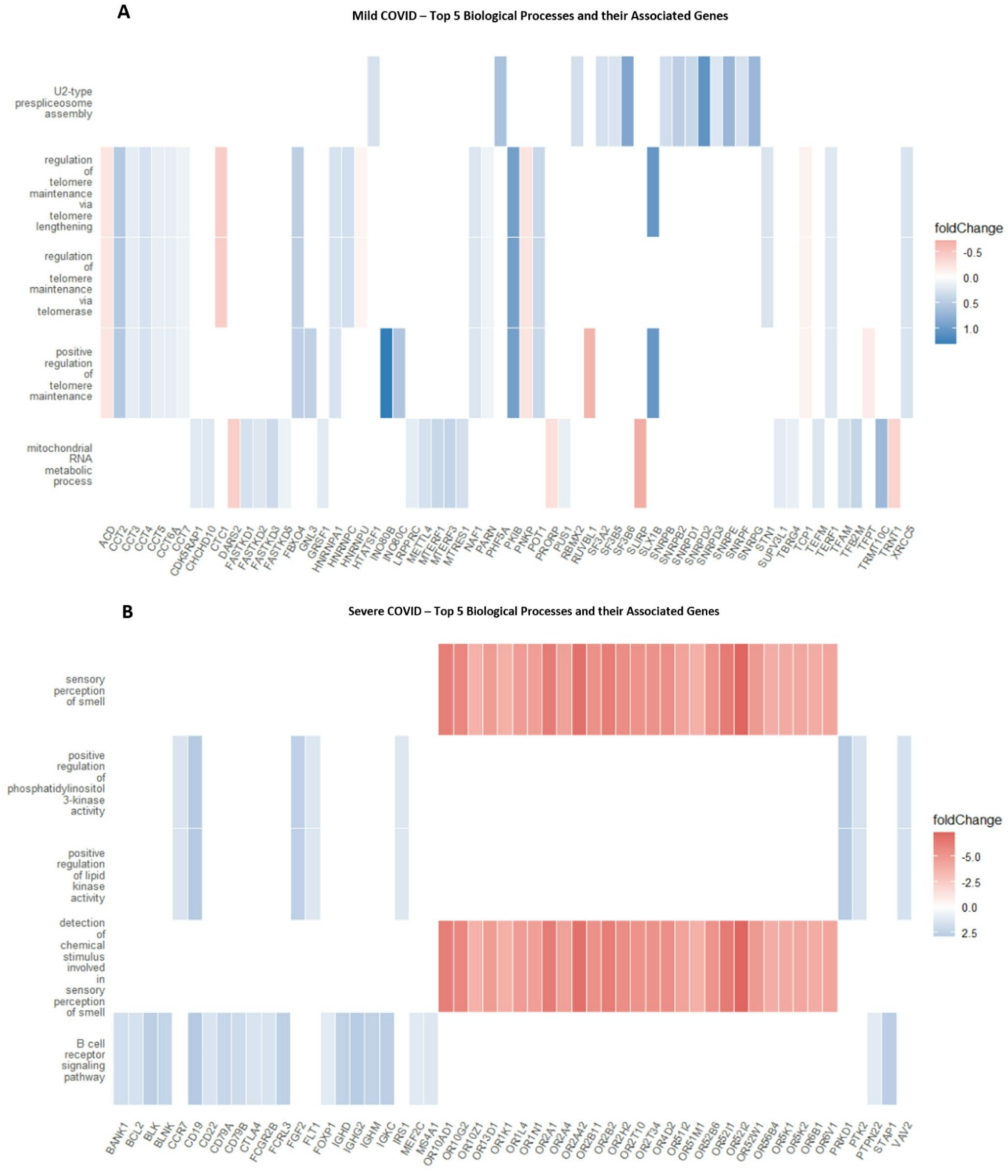
Heat maps showing the top 5 biological processes and their associated genes in recovered COVD-19 patients at 12 weeks post infection compared to controls. The color intensity in each cell of the heatmap reflects the fold change of gene expression, with blue indicating downregulation, red indicating upregulation, and color intensity corresponding to the magnitude of change **A** In Mild COVID, there is range of fold changes across several genes in processes like “U2-type prespliceosome assembly,” “regulation of telomere maintenance via telomere lengthening,” and “mitochondrial RNA metabolic process.” Also, most genes related to telomere maintenance and mitochondrial processes are downregulated (blue), suggesting a reduced activity in these processes in mild cases **B** Severe COVID mostly displayed downregulated gene expression in processes associated with the “detection of chemical stimulus involved in sensory perception of smell” and “B cell receptor signaling pathway.”

Next, we generated GO Enrichment dot plot and heatmaps which revealed distinct gene enrichment pathways associated with different COVID-19 severity levels at 12 weeks post-infection. Figure 8, A-B shows distinct biological processes enriched in recovered mild and moderate COVID-19 patients at 12 weeks post-infection. In mild cases, key processes include telomere maintenance and mitochondrial RNA metabolism, indicating focused cellular repair and genome stabilization. In contrast, moderate cases show broader activation of processes such as ribosome biogenesis, mitochondrial gene expression, DNA repair, and immune activation, reflecting sustained cellular stress. Notably, suppression of the “perception of smell” pathway in moderate cases aligns with reports of persistent anosmia. At 12 weeks post-infection, recovered patients from severe and critical COVID-19 showed persistent alterations in biological processes (Supplementary Figure S4 A-B). In severe cases, key activated pathways included mitochondrial RNA metabolism and lipid signaling, while sensory perception of smell was notably suppressed, consistent with ongoing anosmia. Indeed, it is noteworthy to note that the genes linked to sensory perception of smell and detection of chemical stimulus involved in sensory perception in these patients are heavily downregulated and include a cluster of olfactory receptors (OR) genes such as *OR2AD1*, *OR10G2 OR2B11* and *OR52I2* (Fig. 9B). Critical cases showed strong activation of mitochondrial protein localization, DNA repair, and protein regulation processes, alongside significant suppression of sensory pathways. These findings indicate prolonged disruption of metabolic, repair, and sensory functions in severe and critical COVID-19 recovery.

Remarkably, telomere maintenance processes were observed to be one of the top biological processes activated in patients with mild COVID-19 via activation of genes such as *CCT2*,* CCT3*,* CCT4*,* CCT5* and *CCT6A* (Fig. 9A). Expectedly, we did not observe noticeable differences between the control group and patients who had experienced mild, moderate, severe, or critical COVID-19 at 16wpi or 24wpi.

### COVID-19 exhibits common and tissue-Specific gene expression patterns lung and brain tissue

COVID-19 exhibits diverse clinical manifestations, and to comprehend its impact fully, we explored tissue-specific gene expression patterns between the lungs and the brain given that the infection: (1) predominantly impacts the lungs, manifesting as complex respiratory symptoms, widespread alterations in chest computed tomography (CT) imaging, and a heightened mortality rate associated with pneumonia and (2) manifests in a plethora of neurological symptoms, underscoring the direct or indirect impact of the virus on the brain.

Hence, using lung and brain tissue datasets from GSE150316 and GSE188847 respectively, we compared post-mortem lung and brain tissues from deceased COVID-19 patients to tissues from healthy controls to uncover organ-specific gene expression changes during the acute phase. We expected that this approach will unveil the complexities of COVID-19’s effects on lung and brain tissue while also highlighting common gene pathways impacted by the disease in both. As the first step, we performed differential gene analysis and gene pathway analysis separately each for GSE150316 and GSE188847 to identify DEGs and gene sets enrichment pathways.

**Fig. 10:**
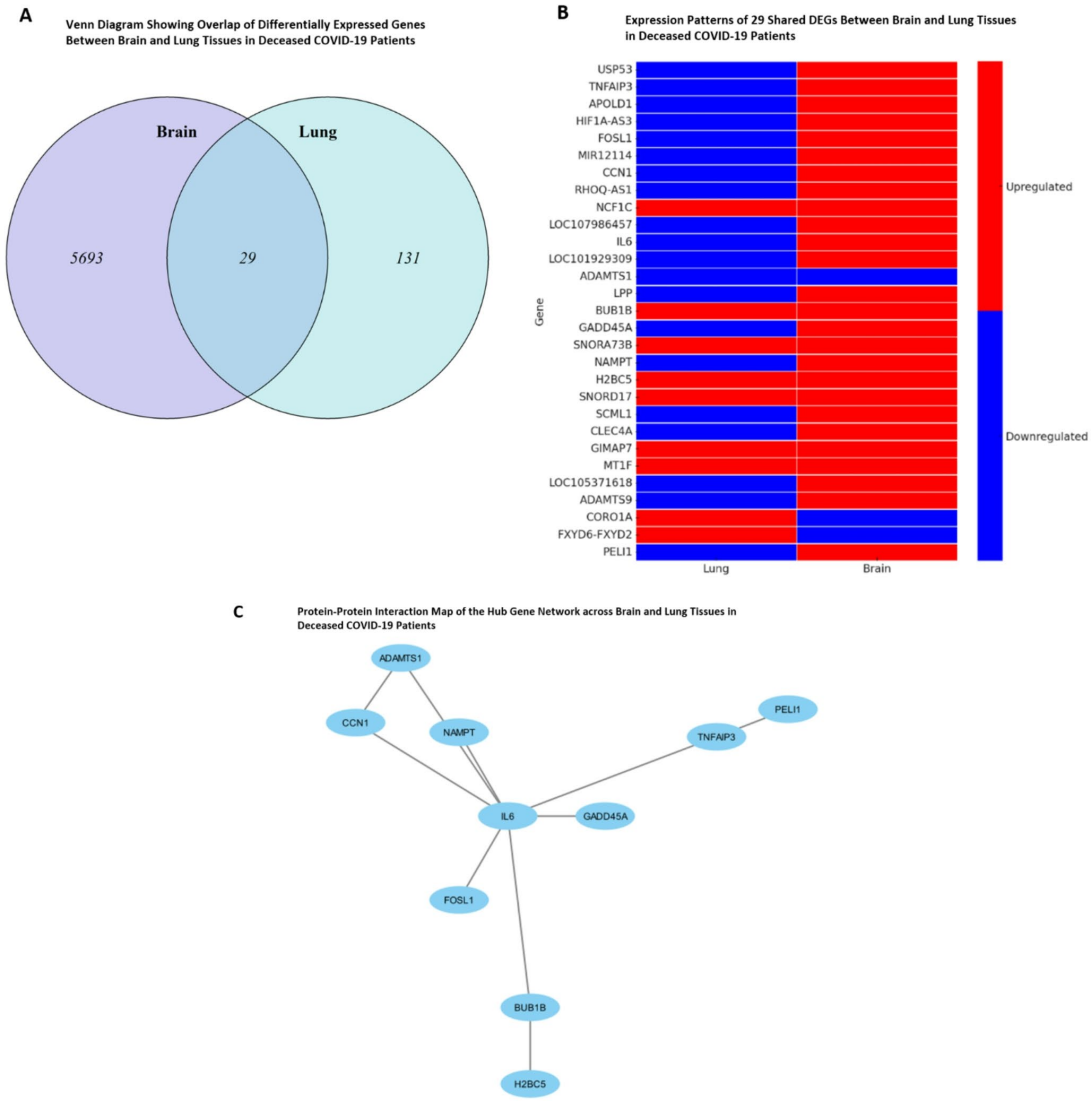
Intersecting DEGs between lung and brain tissue from deceased COVID-19 patients. **A** Shows the number of unique and shared DEGs between the brain (in purple) and lung (in blue) tissues. There are 5693 unique DEGs in the brain, 131 unique DEGs in the lung, and 29 DEGs common to both tissues. **B** Displays the patterns of gene expression for the 29 DEGs shared between lung and brain tissues. Each row represents a gene, while the two columns represent lung and brain tissues. The color coding indicates the level of gene expression, with red representing upregulation and blue indicating downregulation. Most of these shared DEGs are consistently upregulated (red) in lung tissue, while a majority are downregulated (blue) in brain tissue, indicating a potential inverse relationship in gene expression patterns between the two organs in response to COVID-19. **C** Protein–Protein Interaction (PPI) map of the hub genes from Intersecting DEGs between lung and brain tissue from deceased COVID-19 patients. The hub genes are centered around Interleukin 6 (IL6), a cytokine known for its role in the immune response suggesting its significant role in the pathophysiological response in both lung and brain of COVID-19 patients

Next, using “Venn Diagram”, a comparative analysis of the overlapping and unique DEGs between lung and brain tissues was performed. This analysis revealed 29 DEGs are common to both the lungs and brains of individuals affected by COVID-19 (Fig. 10A). In the lung tissue, it was observed that there was a predominant downregulation of cross-tissue DEGs, which included genes such as *USP53*,* TNFAIP3*,* APOLD1*,* HIF1A-AS3*,* FOSL1*, and others (Fig. 10B). These downregulated genes are associated with various biological processes, suggesting an intricate interplay between these processes in the lung’s response to COVID-19. Conversely, in brain tissue, these same DEGs exhibited an upregulated pattern. Genes like *USP53*,* TNFAIP3*,* APOLD1*, and others were notably upregulated (Fig. 10B), indicating a unique gene expression signature in the brain during COVID-19 infection. Our analysis also identified the top 10 intersecting DEGs in brain as *SLC19A2*,* EGR1*,* USP53*,* RAMP2*,* LOC105378179*,* RN7SL2*,* IGHV4-34*,* IGHV3-25*,* IGHV1-18*, and *IGHV1-12*, whilst that for brain tissue were found to be *CORO1A*,* IL6*,* LOC105371618*,* NAMPT*,* BUB1B*,* SCML1*,* GIMA97*,* USP53*,* LPP* and *PELI1*.

Examination of the PPI map revealed from the intersecting genes in Fig. 10B revealed Interleukin-6 as a pivotal hub gene, indicating its substantial role in the immune response within lung and brain tissues of deceased COVID-19 patients (Fig. 10C). Adjacent to IL6, the map highlighted several genes—*ADAMTS1*,* CCN1*,* NAMPT*,* GADD45A*,* FOSL1*,* BUB1B*,* H2BC5*,* TNFAIP3*,* and PELI1*—each connected to *IL6*, suggesting a network of interactions potentially critical to the disease’s pathogenesis.

## Discussion

The ongoing COVID-19 pandemic remains a significant challenge for public health worldwide (Harizi et al. 2021). When the COVID-19 pandemic first emerged, the primary concern was the severe lung damage caused by the SARS-CoV-2 virus. However, it soon became clear that the virus’s impact extends beyond the lungs, affecting multiple organs and tissues including the heart, brain, kidneys, and blood vessels (Clerkin 2020; Raza et al. 2020). In addition, many people who became ill with COVID-19 continue to experience symptoms over long periods of time (National 2020). This research highlights key findings demonstrating the dynamics of disruption for several key gene pathways that persist beyond the COVID-19 recovery phase. Notably, we were able to find commonality in these post-COVID expression perturbations in brain and lung tissue and pathological symptoms observed across both tissue in deceased COVID-19 patients.

Our study gives support to the hypothesis of Singh et al.. (2020) who have previously proposed that the hijacking of host mitochondria by SARS-CoV-2 may be a key mechanism driving the pathogenesis of COVID-19 (Singh et al. 2020). Other studies have also demonstrated a correlation between high SARS-CoV-2 RNA levels in nasopharyngeal and autopsy samples from the heart, kidney, and liver with reduced expression of mitochondrial genes involved in oxidative phosphorylation (OXPHOS) (Guarnieri et al. 2023). In response to the SARS-CoV-2 viral inhibition, host cells may counteract by coordinately upregulating the expression of OXPHOS genes encoded in the nuclear DNA (Singh et al. 2020; Srinivasan et al. 2021). This study demonstrates that there is a persistent increased activation of nuclear-encoded OXPHOS genes namely*COX7C*,* COX7A2*,* ATP5PO*,* ATP5F1C* and *NDUFB8* in blood tissue immediately following COVID-19 recovery, and then a time-dependent tapering off of these genes. Subsequently, we favor a model in which fluctuations in mitochondrial OXPHOS gene expression during the course of acute infection and recovery from COVID-19 results from two influences: the virus’s targeted suppression of mitochondrial DNA genes and the host cell’s compensatory upregulation of nuclear-encoded mitochondrial OXPHOS genes.

Interestingly, over half of the individuals who overcome the initial critical stage of COVID-19 might Post-COVID Syndrome (Chen et al. 2022) and there is emerging and growing body of research implicating exercise intolerance as a significant symptom commonly associated with long COVID. Remarkably, another study has revealed a range of diminished systemic oxygen extraction (EO_2_) among these patients, with a significant number of them demonstrating suboptimal peak aerobic capacity during exercise and elevated protein markers associated with oxidative phosphorylation and glycogen metabolism (Singh et al. 2023). Since in acute stage of COVID-19 infection there is a prevailing inhibition of mitochondrial DNA (mtDNA) gene expression which triggers an over-compensating host activation of nuclear-encoded mitochondrial genes and leads to a compromised mitochondrial-nuclear co-regulation, the sustained upregulation of nuclear-encoded OXPHOS genes as far 24 weeks post infection demonstrated in this study shows consistency to this model and suggests this compensatory host response may still persist post-recovery.

The overexpression of nuclear-encoded mitochondrial genes involved in OXPHOS has been associated with an increased production of reactive oxygen species (ROS) which, in turn, may deteriorate mitochondrial health and further exacerbate oxidative stress in a self-perpetuating vicious cycle in recovered COVID-19 patients. There is also an enhanced ROS formation and oxidative stress in these patients which reduces with attenuation of fatigue symptoms (Medini et al. 2021; Ježek et al. 2018). Also, while an increased*ATP5ME* upregulation promotes ATP production, *NACA*expression ameliorates mitochondrial dysfunction and reduces oxidative stress (Hofmann et al. 2023; Lee et al. 2022; Liang et al. 2021). Some patients who have recovered from the acute phase of COVID-19 continue to experience ongoing hypoxia which is associated with*ATP5ME*downregulation (Liang et al. 2020; Adingupu et al. 2023). The initial stepwise decrease in the levels of*ATP5ME* from 12 to 16 weeks suggest a trend of decreased ATP output regulation, followed by an increase, suggesting an elevated gene expression towards increased ATP production. Hence, this gene expression pattern likely indicates a dynamic host response towards ongoing hypoxic conditions. Expectedly, *NACA* followed a similar pattern, indicating a tandem dipping mitigating response to decreasing ROS associated with ATP energy production at 12–24 weeks, followed by an amplified ameliorating response to ROS production tied to elevated ATP output at 24 weeks.

Previous research also has shown that SARS-CoV-2 infection is negatively correlated with ubiquitin expression and as part of the immune system’s swift response to the SARS-CoV-2 virus invasion there is an elevated expression of ubiquitin in lymphoid morphology. Additionally, enhanced ubiquitination contributes to strengthening immunity against severe SARS-CoV-2 infections (Ryan 2022). Interestingly, mutations in the*UBE3A* gene have been associated with Angelman Syndrome, while additional clinical evidence shows that individuals with this condition may face a heightened risk of severe COVID-19 complications due to inherent mutations affecting their *UBE3A*expression levels— as previously documented in the case of a 14-year-old boy with Angelman Syndrome who experienced severe COVID-19 symptoms, necessitating a 20-day stay in the ICU while showing elevated inflammatory biomarkers (Taha et al. 2022). Although this single case study does not conclusively prove the impact of deficient*UBE3A*expression on COVID-19 outcomes, it represents a remarkable and noteworthy clinical indication, given that teenagers typically exhibit mild or no symptoms of SARS-CoV-2 infection (Taha et al. 2022). Our findings suggest that*UBE3A* expression is upregulated in response to COVID-19 infection, continuing up to 12 weeks post-infection in individuals who have recovered. This observation underscores the protective role of ubiquitination in combating COVID-19. Additionally, at 12 weeks post-infection, patients who experienced critical condition during the acute phase showed relatively lower *UBE3A* gene expression compared to those with severe manifestations, further supporting the critical role of enhanced *UBE3A* expression in influencing the severity of COVID-19 and highlighting the implications for patients with Angelman Syndrome.

Also, our findings also not only demonstrate that *EGFR* is a key gene in the development of critical COVID-19, but it continues to be downregulated at 12 weeks post infection even after recovery. Previous research has demonstrated that the activation of *EGFR*by the SARS-CoV-2 spike protein triggers the phosphorylation of the canonical extracellular signal-regulated kinase1/2 (ERK1/2) and AKT kinase pathway in lung tissue and contributes to the inflammation cascade observed in some COVID-patients (Palakkott et al. 2023; Londres 2022). This suggests that suppressed expression of*EGFR* likely also reflects an excessive activation of the body’s protective defenses against COVID-19, particularly considering the effectiveness of nimotuzumab, an *EGFR*inhibitor, in reducing fibrosis in patients with the disease (Li 2015). In addition, our data suggests*SOX9* as one of the hub genes uniquely down-modulated in critically ill patients as far as 12 weeks post infection. Previous studies have shown that activating *SOX9*is crucial for restoring lung function following acute lung injury (ALI) (Islam and Khan 2020) and is downregulated by the SARS-CoV-2 virus (Reinchisi et al. 2013). Its persistent suppression in patients who have recovered from critical illness, observed up to 12 weeks post-infection, likely signals a prolonged disruption in the reparative mechanisms of pulmonary tissues, and could potentially contribute to the protracted respiratory complications associated with post-acute sequelae of COVID-19 in some patients with severe disease.

Furthermore, this study also identified the unique downregulation of the *SHH* gene at the 12-week mark in recovered critical COVID-19 patients. Contemporary studies have pinpointed the *SHH*gene as vital for the preservation of olfactory and gustatory functions (Miura et al.; Castillo-Azofeifa 2018). This downregulation aligns with emerging evidence suggesting that this gene may be involved in the mechanisms leading to anosmia in COVID-19 patients (Henkin 2021; Lee et al. 2023). Although the precise mechanism remains to be elucidated, there is evidence that it may be initiated by SARS-CoV-2’s 3CL protease, which cleaves septins (SEPT2,−6, and − 9) in ciliated cells (Amin et al. 1999). This action results in an excess of cleaved fragments that systematically obstruct cellular processes, thereby disrupting*SHH*expression (Götschel et al. 2013). While is evidence of cross-talk between the*EGFR* and *SHH*signaling pathways (Reinchisi et al. 2013), the nature of this interaction depends on the specific mechanisms of cross-talk in the cells affected. Hence, though the persisting downregulation of both*SHH* and *EGFR* in critically ill COVID demonstrate common expression direction for both, additional research is needed to clarify the exact mechanism by which both genes interact in COVID-19 in tissues other than blood.

Interleukin 6 (*IL6*) has been recognized to be the most important driver of immune dysregulation and ARDS in COVID-19 and a predictive factor for a severe form of the disease (Magro 2020; Giamarellos-Bourboulis et al. 2020; Setyo Nugroho et al. 2022; Zhang et al. 2020; Mandel et al. 2020). Interestingly, our study also identified interleukin-6 as a central hub within the lung and brain tissues of deceased COVID-19 patients and was upregulated in the latter tissue but unexpectedly downregulated in the former. Contemporary research has established that elevated IL6 expression is a hallmark in the pulmonary tissue of COVID-19 patients (Han et al. 2020). Our study, while seemingly at odds with this prevailing scientific consensus, may indicate a tailored tissue-specific response in the lungs and brain following COVID infection. In early COVID-19, the initial host response in the lung involves the release of large amounts of cytokines, including interleukin-6 (Santa Cruz et al. 2021). This is followed by an anti-inflammatory negative feedback mechanism in which attenuates the interleukin-6 response. Given that IL6 downregulation is associated with a less disease severity and its upregulation signals the opposite our findings likely indicate a downgraded interleukin-6 response in the lung and heightened response in the brain in deceased patients. This either demonstrates stepwise approach to controlling interleukin-6-induced cytokine storm in the host that is more robust in the lungs and less so in the brain reflect a differential temporal interleukin-6 response between these two critical organs or the confounding effect of therapeutic interventions applied to treat pulmonary symptoms in the patient population. Exploring whether this IL6 suppression is a strategically-orchestrated adaptation or a consequence of clinical interventions may be a worthwhile endeavor for future research. Additionally this study highlights the brain as heavily impacted target of the interleukin-6 response and underscores this cytokine’s pivotal role in mediating neuroinflammatory processes. RemarkablySenchenkova et al. (2019) have previously not only demonstrated a positive association between interleukin-6 levels to increased immature platelet production and thrombosis but have also shown that a reduction in levels of IL-6 results in a reduction of thrombotic complications (Senchenkova et al. 2019). Interestingly, cerebral venous system thrombosis, ischemic stroke, white matter irregularities, hemorrhagic lesions, and perfusion irregularities remain key physiological feature that are frequently observed in COVID-19 patients (Cavalcanti et al. 2020; Taha et al. 2022). IL6 upregulation in the brain therefore is consistent with the cytokine ‘storm’ observed in COVID-19 and consequently plays a critical role in driving the thrombotic pathology observed in COVID-19 patients’ brain, and can be potentially be targeted for ameliorating this risk in patients. One of the key pathogenic features of a COVID-19 infected lung tissue is widespread fibrosis (Valdebenito et al. 2021). This study also revealed*RAMP2* as one of the top 10 genes uniquely upregulated in the lung tissue of COVID-19 patients. *RAMP2*functions by associating with the adrenomedullin receptor, thereby enhancing its sensitivity or increasing its expression (Kamitani et al. 1999). Previous research has shown that adrenomedullin maintains vascular tone as well as endothelial barrier function and is markedly increased during severe inflammatory disorders such as sepsis, pneumonia, and COVID-19 (Kita and Kitamura 2022). Based on how*RAMP2* affects adrenomedullin, this study demonstrates that the *RAMP2* upregulation observed in the lung tissue of COVID-19 infected patients is most likely a compensatory response to lung injury caused by COVID-19, and is aimed at mitigating damage and inflammation.

Ackermann et al. (2020) have demonstrated three distinctive angiocentric features in the lungs of patients with COVID-19: severe endothelial injury, widespread blood clotting with diseased capillaries and significant new vessel growth. Additionally, they showed that the severe endothelial injury is caused by damage to the endothelial cells, while the significant new vessel growth is likely a result of the body’s response to the virus – a response known as intussusceptive angiogenesis (IA), reflecting the body’s attempt to compensate for the clotting and blood vessel damage (Ackermann et al. 2020). Interestingly our study revealed “vasculature development” as among the top 10 biological processes uniquely suppressed in the lungs of deceased COVID-19 patients. While this finding contrasts withAckermann et al. (2020) study, we believe this could be attributed to the effect of therapeutic administered as part of modulating the symptoms in the patients from which the samples for GSE150316 were obtained. Among the therapeutic strategies implemented in COVID-19, a few are known to inhibit angiogenesis, including glucocorticoids and bevacizumab which both of which been used as an add-on therapy in COVID-19 to modulate intussusception (Martin et al. 2009; Pang et al. 2021). Though there is no available metadata showing the patients in our sample were subjected to anti-vasculature therapeutic agents, nonetheless, these findings are promising and highlight the need for future studies focusing on treatment-naïve COVID-19 infected lung tissue.

This study provides a comprehensive transcriptomic analysis across blood, lung, and brain tissues from recovered and deceased COVID-19 patients using publicly available RNA-seq datasets. By applying robust bioinformatics methods, we identified persistent alterations in gene expression, including long-term dysregulation of mitochondrial genes, differential expression patterns shaped by initial disease severity, and tissue-specific signatures such as elevated *SHH* expression linked to olfactory dysfunction and central *IL6* signaling in lung and brain inflammation. These findings offer novel insights into the prolonged molecular impact of SARS-CoV-2 infection and highlight pathways that may inform targeted therapeutic strategies for individuals experiencing post-acute sequelae of COVID-19.

While our findings provide valuable insights, several limitations should be acknowledged. First, the analyses relied on bulk RNA-seq data, which limits the ability to resolve gene expression at the single-cell level. Thus, observed transcriptional changes may reflect shifts in cell population proportions rather than changes within specific cell types. Second, the relatively small sample sizes for lung (*n* = 10) and brain (*n* = 24) tissues may reduce statistical power and increase the likelihood of false negatives. However, these samples represent rare and valuable postmortem datasets, offering critical insights into organ-specific COVID-19 responses. Third, the brain tissue dataset (GSE188847) is listed as unpublished in GEO as of 2021. Despite this, the dataset is publicly available and was rigorously analyzed using standardized pipelines. Finally, while we observed associations between gene expression and clinical severity or recovery timepoints, direct pathological correlations at the individual patient level were not feasible due to limited metadata availability.

## Conclusion

To conclude, our study provides a comprehensive understanding of the intricate interplay between viral action and host response in COVID-19, highlighting the complex dynamics of gene expression in blood, lung and brain tissue. SARS-CoV-2 viral inhibition of mitochondrial function in patients occurs during acute infection and host cells may counteract by coordinately upregulating the expression of OXPHOS genes encoded in the nuclear DNA. COVID-19 induces significant mitochondrial dysfunction, leading to the upregulation of nuclear-encoded OXPHOS genes. The uptick in these genes is an adaptive response to compensate for energy deficits by bolstering mitochondrial respiratory efficiency. Though the compensatory mechanism remains active several weeks after recovery, its intensity reduces gradually as the recovered patient accrues more post-recovery days. Moreover, one of the key COVID-19-induced compensatory gene pathways include the enhanced expression of *UBE3A* which drives a ubiquitin-modulated host response to COVID-19 and is positively associated with reduced disease severity. Consequently, Angelman Syndrome patients who naturally have mutations in the *UBE3A* gene might be at an elevated risk for experiencing more severe forms of COVID-19. Also, *IL6* expression remains is a crucial immune process that is central to the pathophysiology of COVID-19 in lung and brain tissue, particularly in the latter where plays a role in exacerbating hemorrhagic and thrombotic events. Nevertheless, additional research is needed to understand the temporal dynamics of its expression from infection onset to disease progression in COVID-19 patients. In summary, this study not only advances our knowledge of the long-term effects of COVID-19 but also opens up tailored treatment strategies for COVID-19 survivors as well as new avenues for targeted interventions ameliorating pulmonary and neurological symptoms in acute settings.

## Supplementary Information

Below is the link to the electronic supplementary material.

Supplementary Material 1

Supplementary Material 2

## Data Availability

No datasets were generated or analysed during the current study.
