# Supplementary material for: Peripheral blood, lung and brain gene signatures in recovered and deceased patients with COVID-19
Source: In Silico Pharmacol. 2025 Oct 22;13(3):159. doi: 10.1007/s40203-025-00450-1 (PMC12545983; doi:10.1007/s40203-025-00450-1)
Supplement: Supplementary file 2 — Supplementary Material 2 [file 40203_2025_450_MOESM2_ESM.pptx]

## Slide 1
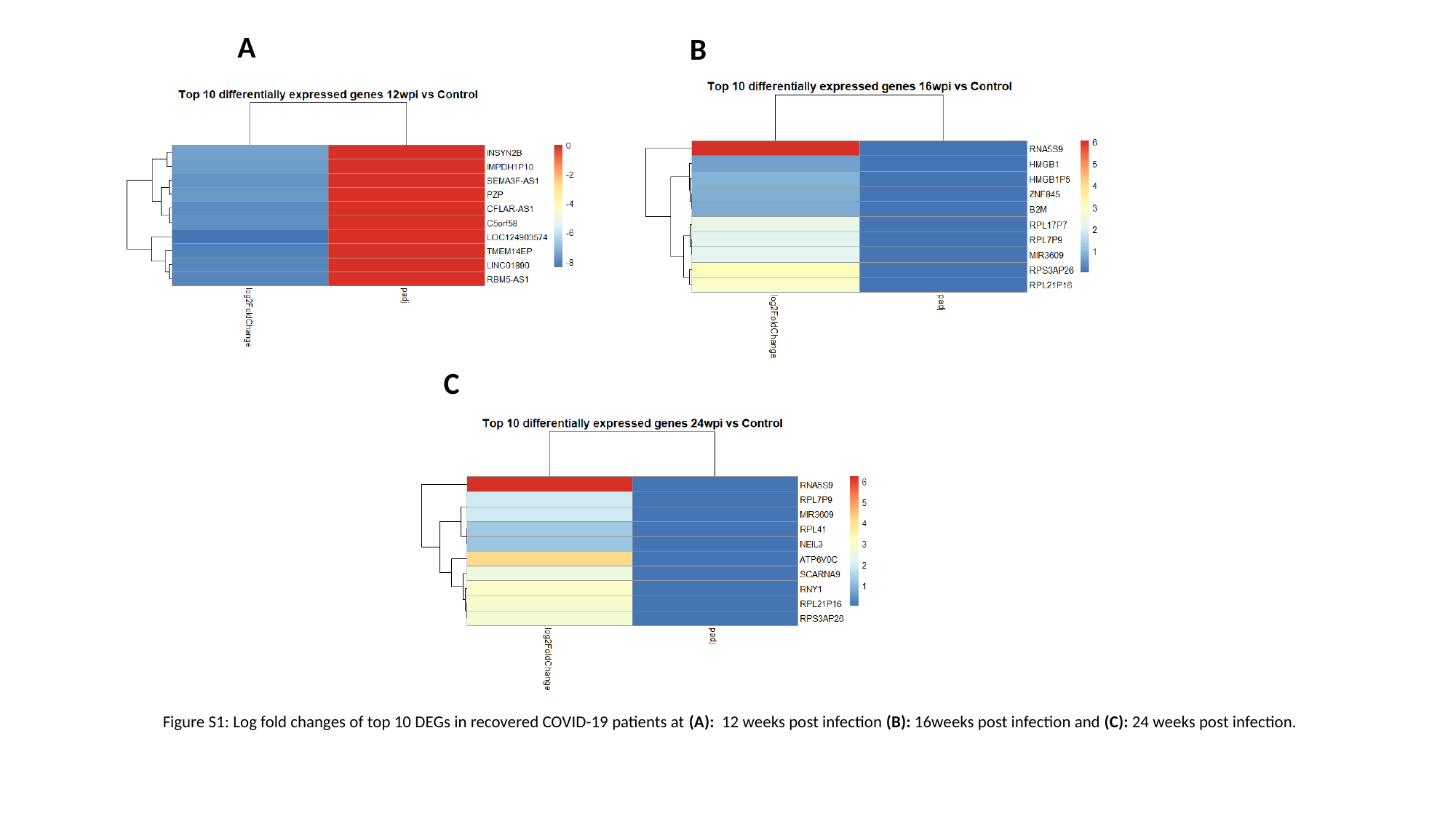

A
B
C
Figure S1: Log fold changes of top 10 DEGs in recovered COVID-19 patients at (A): 12 weeks post infection (B): 16weeks post infection and (C): 24 weeks post infection.

## Slide 2
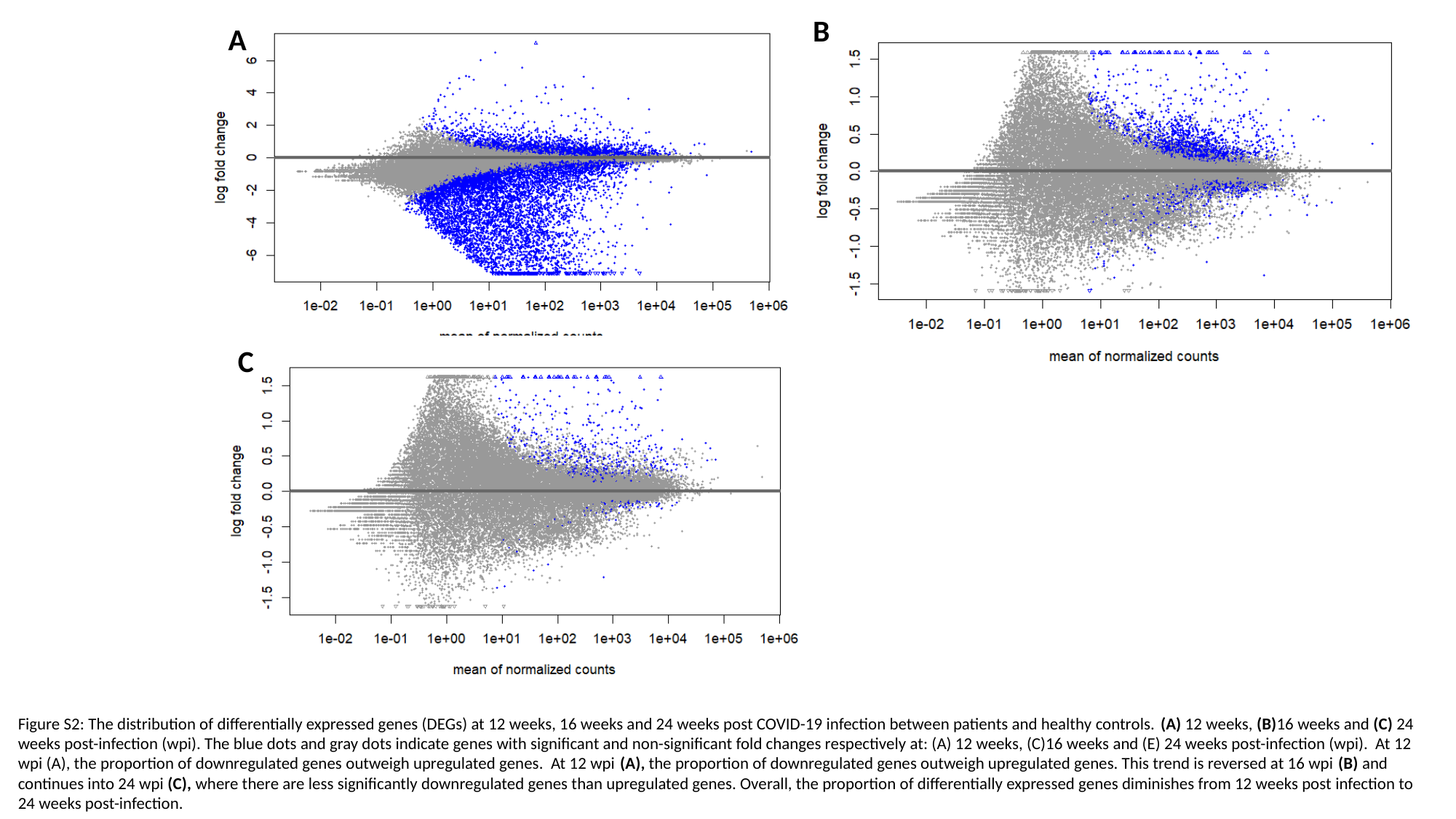

B
A
C
Figure S2: The distribution of differentially expressed genes (DEGs) at 12 weeks, 16 weeks and 24 weeks post COVID-19 infection between patients and healthy controls. (A) 12 weeks, (B)16 weeks and (C) 24 weeks post-infection (wpi). The blue dots and gray dots indicate genes with significant and non-significant fold changes respectively at: (A) 12 weeks, (C)16 weeks and (E) 24 weeks post-infection (wpi). At 12 wpi (A), the proportion of downregulated genes outweigh upregulated genes. At 12 wpi (A), the proportion of downregulated genes outweigh upregulated genes. This trend is reversed at 16 wpi (B) and continues into 24 wpi (C), where there are less significantly downregulated genes than upregulated genes. Overall, the proportion of differentially expressed genes diminishes from 12 weeks post infection to 24 weeks post-infection.

## Slide 3
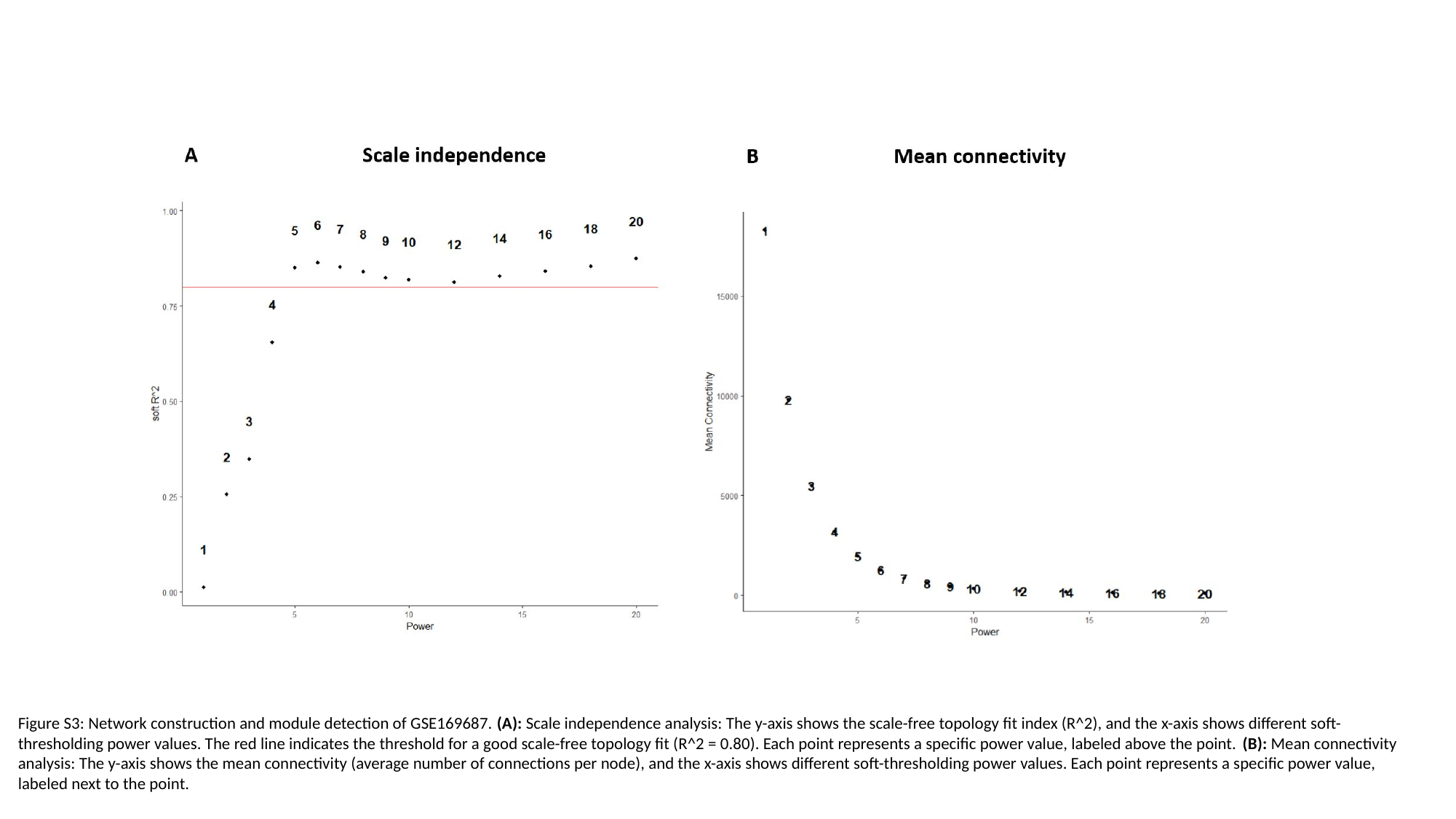

Figure S3: Network construction and module detection of GSE169687. (A): Scale independence analysis: The y-axis shows the scale-free topology fit index (R^2), and the x-axis shows different soft-thresholding power values. The red line indicates the threshold for a good scale-free topology fit (R^2 = 0.80). Each point represents a specific power value, labeled above the point. (B): Mean connectivity analysis: The y-axis shows the mean connectivity (average number of connections per node), and the x-axis shows different soft-thresholding power values. Each point represents a specific power value, labeled next to the point.

## Slide 4
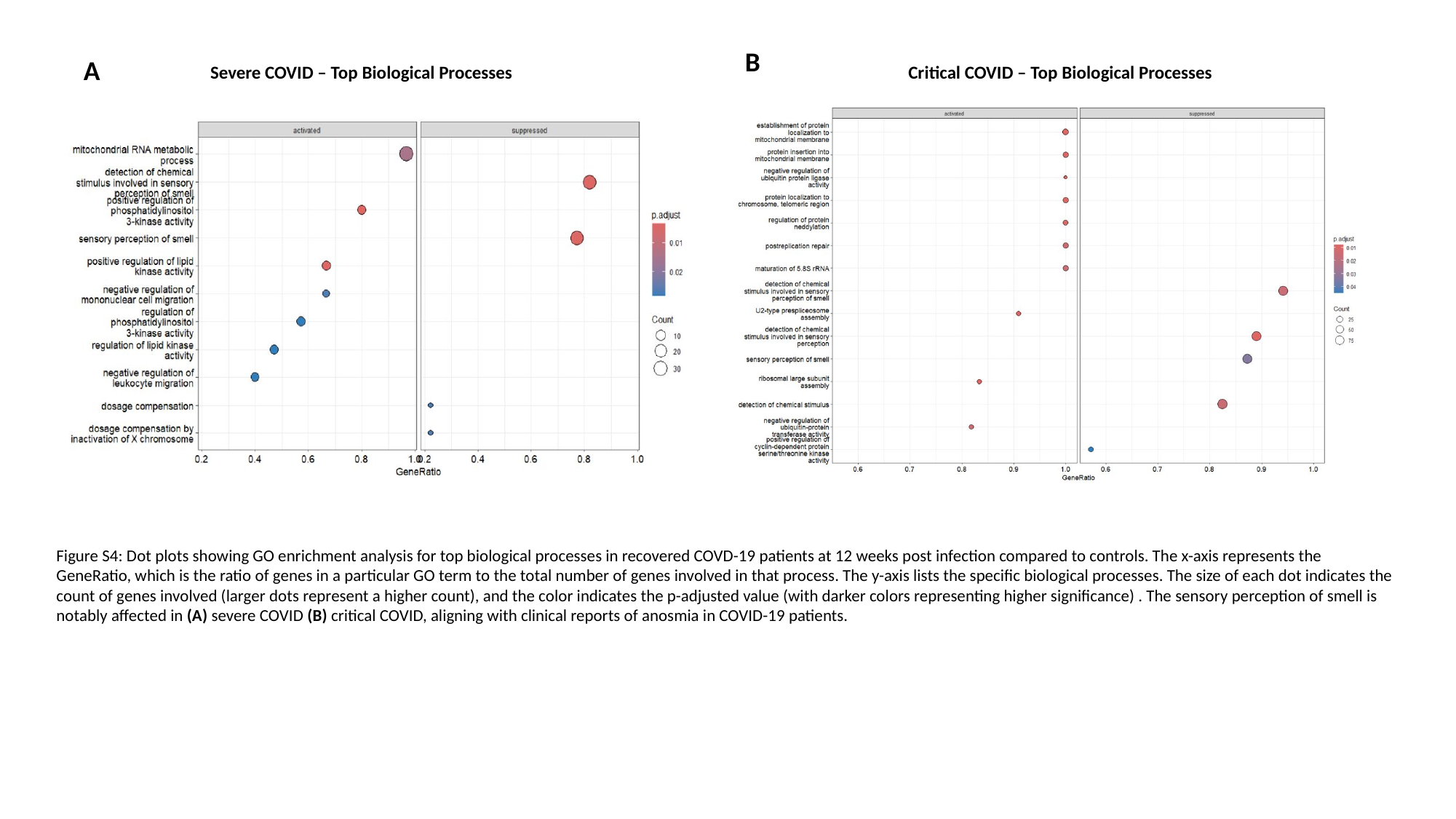

B
A
Severe COVID – Top Biological Processes
Critical COVID – Top Biological Processes
Figure S4: Dot plots showing GO enrichment analysis for top biological processes in recovered COVD-19 patients at 12 weeks post infection compared to controls. The x-axis represents the GeneRatio, which is the ratio of genes in a particular GO term to the total number of genes involved in that process. The y-axis lists the specific biological processes. The size of each dot indicates the count of genes involved (larger dots represent a higher count), and the color indicates the p-adjusted value (with darker colors representing higher significance) . The sensory perception of smell is notably affected in (A) severe COVID (B) critical COVID, aligning with clinical reports of anosmia in COVID-19 patients.

## Slide 5
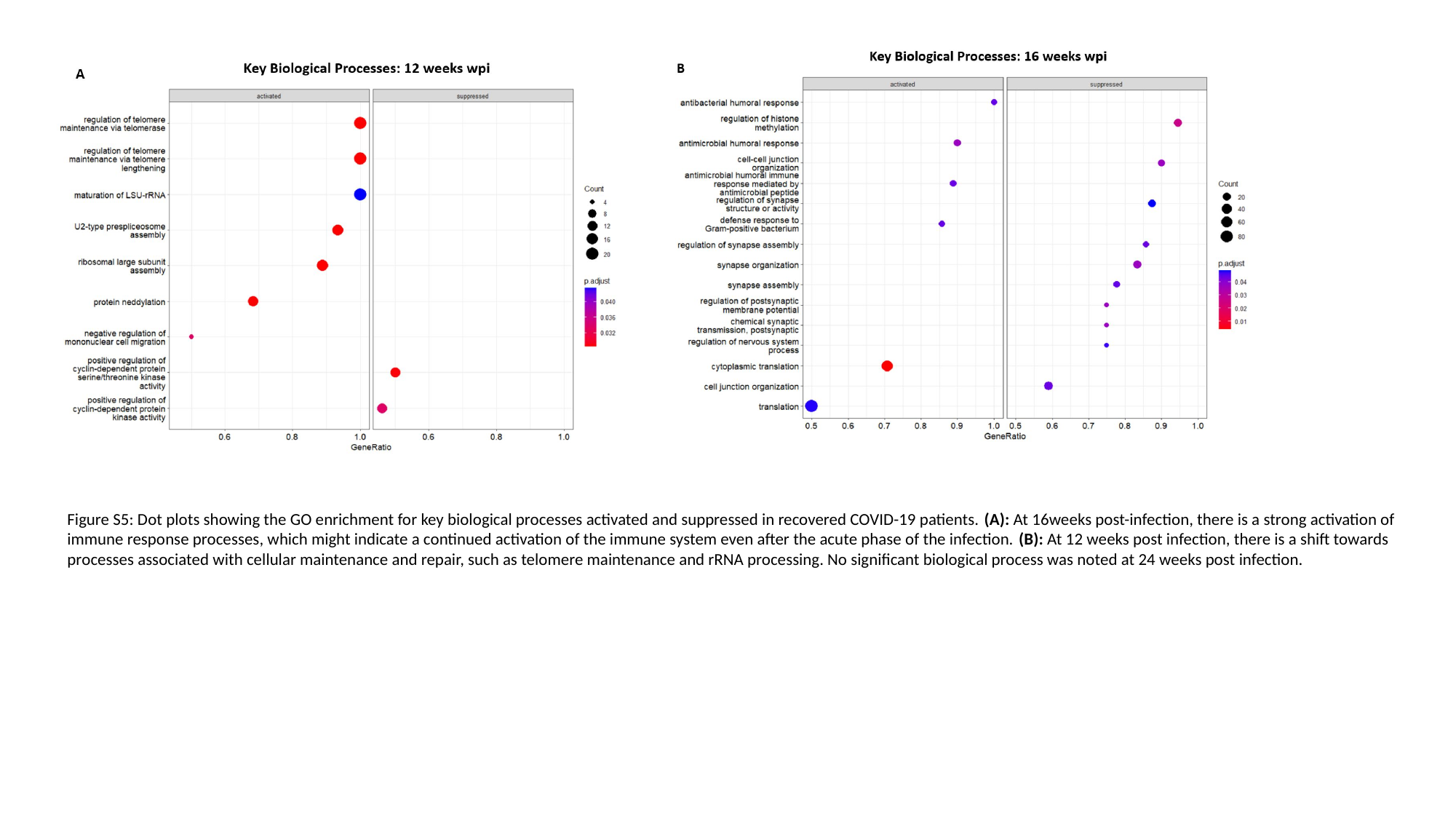

Figure S5: Dot plots showing the GO enrichment for key biological processes activated and suppressed in recovered COVID-19 patients. (A): At 16weeks post-infection, there is a strong activation of immune response processes, which might indicate a continued activation of the immune system even after the acute phase of the infection. (B): At 12 weeks post infection, there is a shift towards processes associated with cellular maintenance and repair, such as telomere maintenance and rRNA processing. No significant biological process was noted at 24 weeks post infection.
